# Genes required for phosphosphingolipid formation in *Caulobacter crescentus* contribute to bacterial virulence

**DOI:** 10.1371/journal.ppat.1012401

**Published:** 2024-08-02

**Authors:** Roberto Jhonatan Olea-Ozuna, Sebastian Poggio, Ed Bergström, Aurora Osorio, Temidayo Oluyomi Elufisan, Jonathan Padilla-Gómez, Lourdes Martínez-Aguilar, Isabel M. López-Lara, Jane Thomas-Oates, Otto Geiger

**Affiliations:** 1 Centro de Ciencias Genómicas, Universidad Nacional Autónoma de México, Avenida Universidad s/n, Cuernavaca, Morelos, Mexico; 2 Departamento de Biología Molecular y Biotecnología, Instituto de Investigaciones Biomédicas, Universidad Nacional Autónoma de México, Mexico City, Mexico; 3 Centre of Excellence in Mass Spectrometry and Department of Chemistry, University of York, Heslington, York, United Kingdom; Ruhr University Bochum, GERMANY

## Abstract

Sphingolipids are ubiquitous in membranes of eukaryotes and are associated with important cellular functions. Although sphingolipids occur scarcely in bacteria, for some of them they are essential and, in other bacteria, they contribute to fitness and stability of the outer membrane, such as in the well-studied α-proteobacterium *Caulobacter crescentus*. We previously defined five structural genes for ceramide synthesis in *C*. *crescentus*, among them the gene for serine palmitoyltransferase, the enzyme that catalyzes the committed step of sphingolipid biosynthesis. Other mutants affected in genes of this same genomic region show cofitness with a mutant deficient in serine palmitoyltransferase. Here we show that at least two phosphosphingolipids are produced in *C*. *crescentus* and that at least another six gene products are needed for the decoration of ceramide upon phosphosphingolipid formation. All eleven genes participating in phosphosphingolipid formation are also required in *C*. *crescentus* for membrane stability and for displaying sensitivity towards the antibiotic polymyxin B. The genes for the formation of complex phosphosphingolipids are also required for *C*. *crescentus* virulence on *Galleria mellonella* insect larvae.

## Author summary

Sphingolipids participate in the formation of biological membranes and molecular signaling in higher organisms. Many bacteria also accommodate sphingolipids in their membranes. Here we report that eleven genes participate in the synthesis of complex bacterial phosphosphingolipids. Our data show that these lipids contribute to membrane stability, but also confer sensitivity towards certain antibiotics. The bacterium *Caulobacter crescentus* is widely distributed in freshwater lakes and streams and was considered to be non-virulent. However, it has recently been shown that *C*. *crescentus* can be virulent in certain model systems and we demonstrate that complex phosphosphingolipids are important for its virulence.

## Introduction

Sphingolipids (SphLs) are essential ingredients of eukaryotic membranes and participate in membrane structural organization, lipid raft formation, cell signaling, and many other important cellular processes [1]. Although SphLs were considered to be rare components of bacterial membranes, they are more widespread than previously thought [2]. SphLs seem to be located mainly in the outer layer of the outer membrane (OM) in diderm bacteria and are encountered in many members of the Proteobacteria and the Bacteroidetes [3,4]. The outer layer of the OM is usually composed of lipopolysaccharides (LPSs) which provide an efficient barrier against external toxins and antibiotics [5]. As LPSs are partially or completely replaced by SphLs in some bacteria, SphLs are expected to also serve as functional replacements for LPSs [6,7].

The *de novo* biosynthesis of SphLs in eukaryotes takes place in five stages [1]. However, in bacteria only the first step was known, in which serine is condensed with a fatty acyl-thioester to form 3-oxo-sphinganine (3-ketosphinganine) catalyzed by serine palmitoyltransferase (Spt). While Spts of the Bacteroidetes use the thioester fatty acyl-coenzyme A (acyl-CoA) as substrate, like Spts of eukaryotes, recent work clarified that Spts from α-, β-, and γ-Proteobacteria (summarized as *Rhodobacteria*) prefer an acylated specialized acyl carrier protein (AcpR) over acyl-CoA as substrate [8]. The substrate for this reaction, acyl-AcpR, was shown to be formed by a specialized acyl-ACP synthetase AasR [8,9]. AasR and AcpR are predicted to be localized in the cytoplasm. In the α-proteobacterium *Caulobacter crescentus* two more structural genes necessary for ceramide formation were identified [9], one potentially encoding a dehydrogenase (CC_1164) and the other an *N*-acyltransferase (CC_1154). In analogy to eukaryotic ceramide synthesis, we proposed that CC_1164 might be the dehydrogenase (CerR) that reduces 3-oxo-sphinganine to sphinganine and that CC_1154 might be the *N*-acyltransferase (CerS) that converts sphinganine to *N*-acyl-sphinganine (dihydroceramide) [9]. A more recent study suggests that the order of these last two steps for dihydroceramide formation might be inverted in *C*. *crescentus* [2]. Another study indicates that Spt and CerS are localized in the cytoplasm, whereas CerR is found in the periplasmic space [10]. CerS and CerR seem to be periferically associated with the inner membrane (IM) [10]. However, to date it is not clear how an NADH-dependent CerR might be able to reduce oxidized ceramide to dihydroceramide in a NADH-free periplasmic environment. The bacterial CerS and CerR enzymes seem to lack eukaryotic orthologs and inhibitors directed against these enzymes might be able to block bacterial ceramide synthesis selectively.

Besides *de novo* ceramide biosynthesis, eukaryotes have a SphL recycling or salvage pathway in which sphingosine, previously generated by SphL degradation, is reacylated by ceramide synthase to yield ceramide [11]. It is estimated that more than half of the ceramide synthesis capacity in eukaryotes is contributed by this salvage pathway. The field of membrane lipid recycling in bacteria has long been neglected [12] and although it is presently not clear whether a sphingolipid salvage pathway exists in bacteria, it is noteworthy that the bacterial *cerS* gene always belongs to a different operon, and therefore transcriptional unit, than the genes required for *de novo* ceramide synthesis only (*aasR*, *cerR*, *acpR*, *spt*) [2,8,9]. As CerS would be required for both, *de novo* ceramide biosynthesis and the salvation pathway, its relative amount with regard to the proteins of *de novo* synthesis only would have to be increased, if salvage pathway-derived sphingosine would have to be reacylated as well.

*C*. *crescentus* is able to produce at least two different groups of complex SphLs, glyco-SphLs (GSphLs) and phospho-SphLs (PSphLs). Under phosphate-limiting growth conditions, GSphLs are formed by the action of two glycosyl transferases [13]. For the formation of the PSphL ceramide di-phosphoglycerate three genes were reported to be required [14].

LPSs as well as bacterial SphLs are synthesized at the IM of diderm bacteria. However, their final destination seems to be the outer layer of the OM. Transport of LPSs from the IM to the OM has been intensely studied. After assembly of the core lipid A structure in the inner layer of the IM, the MsbA transporter flips the lipid A to the outer layer of the IM where it is further modified [15]. LPS is then transported through the cell envelope by the LPS transport (Lpt) complex consisting of seven essential Lpt proteins [16]. Initially, the ATP-driven LptB_2_FGC ABC transporter extracts LPS from the IM. Then LPS is positioned onto a transenvelope bridge involving LptF, LptG, LptC, LptA, and LptD. LPSs are pushed across the bridge until they reach the LptDE translocon which accommodates LPS in the outer layer of the OM [17]. Presently it is not known how SphLs are transported from the IM to the OM.

For some SphL-forming bacteria that lack LPS, SphL synthesis seems to be essential as exemplified in the case of *Sphingomonas koreensis* [9,18]. In bacteria that form both LPSs and SphLs, phenotypes of mutants deficient in SphLs are more subtle than in bacteria devoid of LPSs. In the case of *C*. *crescentus*, survival at elevated cultivation temperatures is dramatically reduced in mutants that cannot produce SphLs when compared to the wild type [9]. Also, SphL-deficient mutants are much more sensitive to detergent treatment than the wild type [9]. Surprisingly, SphL-deficient mutants are more resistant than the wild type to the antibiotic polymyxin B [9,13,18], maybe due to the fact that they cannot synthesize the SphLs, which might be the target molecule of polymyxin B in *C*. *crescentus*.

As most bacterial isolates of *Caulobacter* have been isolated from freshwater sources, this genus was considered to be non-virulent. However, clinical *Caulobacter* isolates have been described [19,20]. A recent report [21] shows that both clinical and non-clinical isolates of *Caulobacter* show virulence on greater wax moth (*Galleria mellonella*) larvae due to a heat-resistant, cell-associated factor.

Here we show that in addition to the five genes required for ceramide synthesis, another six genes are needed for PSphL synthesis. Mutants in all of these eleven genes are impaired in membrane stability as they are sensitive to the detergent deoxycholate, but at the same time are much more resistant to polymyxin B than the wild type. We also show that intact genes for PSphL synthesis are required for *C*. *crescentus* virulence on *G*. *mellonella* larvae.

## Results and discussion

### Serine palmitoyltransferase required for the biosynthesis of phosphosphingolipids

**Fig 1:**
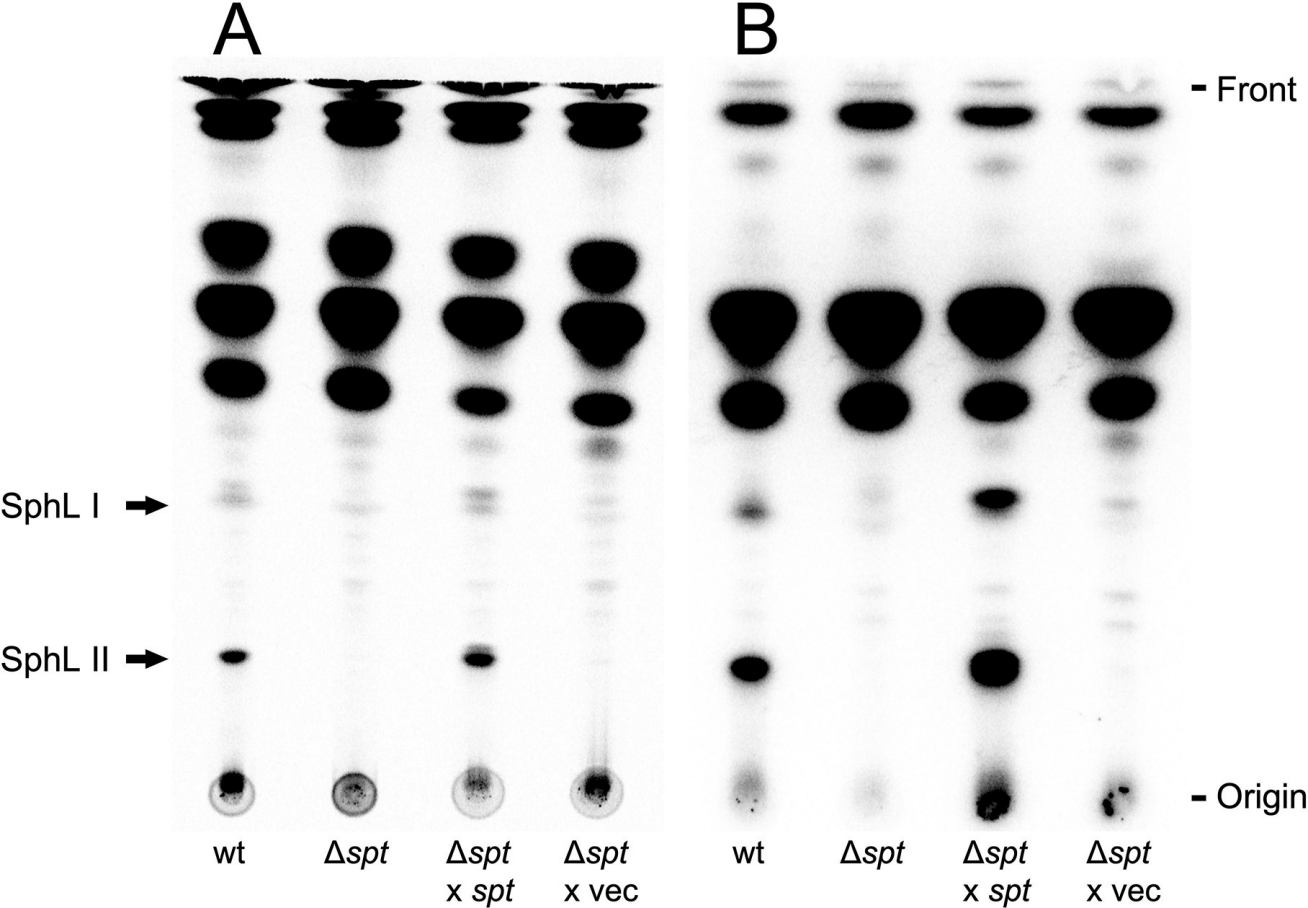
Formation of two phosphosphingolipids in *C*. *crescentus* requires the gene for serine palmitoyltransferase (*spt*). Different strains of *C*. *crescentus* [wild type strain (wt), *spt*-deficient mutant (Δ*spt*), *spt*-deficient mutant harboring the *spt* gene *in trans* (Δ*spt* x *spt*), and *spt*-deficient mutant harboring the empty vector pRXMCS-2 (Δ*spt* x vec)] were cultivated on complex medium in the presence of ^14^C-acetate (A) or ^32^P-phosphate (B). For strains harboring the pRXMCS-2 vector or derivatives of it, xylose and kanamycin were added to the culture media. After harvesting cells, lipids were extracted, lipid samples were separated in chloroform/methanol/acetic acid/water (8:3:2:1) by TLC and developed chromatograms were analyzed by phosphorimaging. Arrows indicate potential PSphLs (SphL I and SphL II) formed by *C*. *crescentus*.

Previous studies revealed that Spt is required for the formation of ceramide and GSphLs [9,13]. Applying different thin-layer chromatographic (TLC) separations, we explored whether we were able to detect phosphorus-containing lipids that were formed in a Spt-dependent way. Separation of ^14^C-acetate-labeled lipids revealed that two lipids (SphL I and SphL II) were formed in *C*. *crescentus* wild type, that were not detected in the *spt*-deficient mutant (Fig 1A). Complementation of the *spt*-deficient mutant with a plasmid containing the intact *spt* gene restored the formation of SphLs I and II, which was not the case when the *spt*-deficient mutant harbored an empty vector instead (Fig 1A). When the same strains were radiolabeled with ^32^P-phosphate, TLC analyses of lipid samples indicate that SphLs I and II contain phosphorus and are formed in the wild type but not in the *spt*-deficient mutant (Fig 1B). Therefore, as SphLs I and II require Spt for their formation, they must be PSphLs. Again, the *spt*-deficient mutant with the intact *spt* gene *in trans* formed ^32^P-labeled SphLs I and II, which was not the case when the *spt*-deficient mutant contained the empty vector (Fig 1B). These data suggest that in *C*. *crescentus* at least two different PSphLs are formed, SphL I and SphL II. Upon TLC separation, SphL I as well as SphL II may appear as double spots, respectively, and these might be the hydroxylated (slower migrating) and non-hydroxylated (faster migrating) versions of PSphls detected in our subsequent mass spectrometric studies.

### Ceramide phosphoglycerate formation in *C*. *crescentus* is serine palmitoyltransferase-dependent

In a recent study [14], ceramide phosphate, ceramide phosphoglycerate (CPG), and ceramide di-phosphoglycerate (CPG2) were reported as PSphLs formed in *C*. *crescentus*. Based on these results, Zik and colleagues proposed a model for PSphL biosynthesis in *C*. *crescentus* which involved CpgB (CC_1160) as a ceramide kinase, converting ceramide to ceramide phosphate, CpgC (CC_1161) converting ceramide phosphate to ceramide phosphoglycerate, and CpgA (CC_1159) converting ceramide phosphoglycerate to ceramide containing two phosphoglycerate moieties [14].

**Fig 2:**
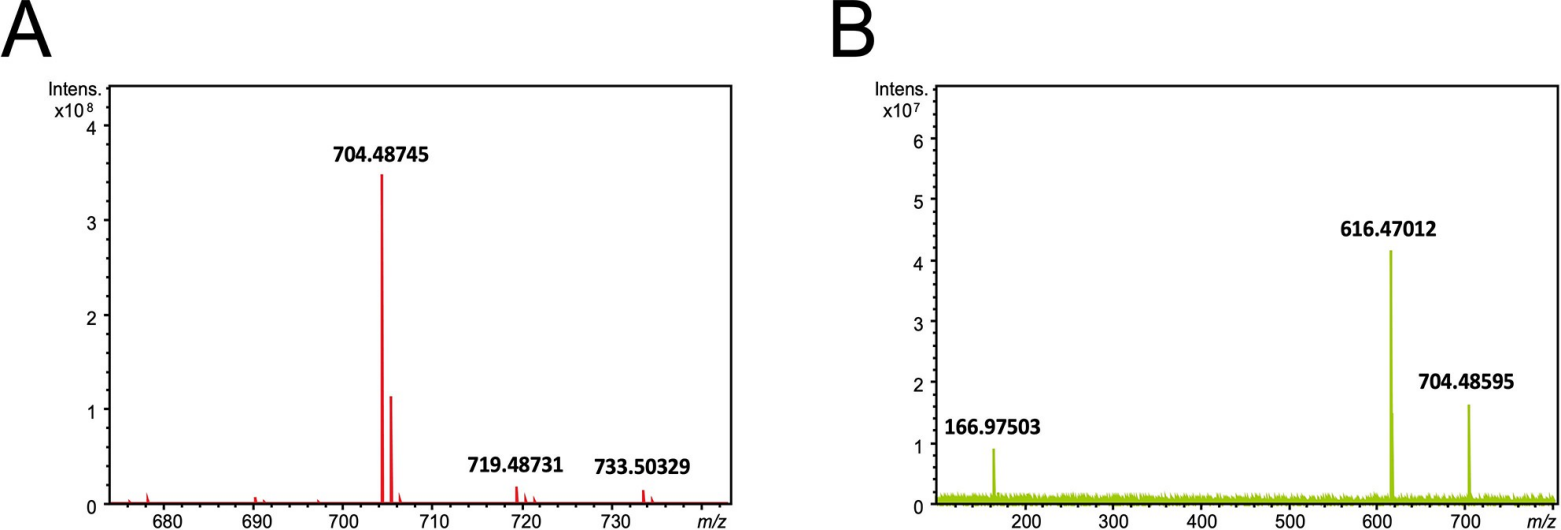
Negative ion mode ESI-FT-ICR mass spectrum of lipid extract of *C*. *crescentus spt*-deficient mutant expressing *spt in trans*, showing intense M-H^-^ signal at *m/z* 704.48745 (A). Negative ion mode product ion spectrum obtained on collision inducted dissociation of the M-H^-^ precursor ion at *m*/*z* 704.48745 (B).

On mass spectrometric analysis, our lipid extracts of an *spt*-deficient mutant that expressed *spt in trans* gave a very intense signal (*m/z* 704.48585) in the negative ion mode electrospray ionization Fourier-transform ion cyclotron resonance (ESI-FT-ICR) mass spectrum (Fig 2A), which was not detected in such spectra of lipid extracts of an *spt*-deficient mutant harboring an empty vector. The signal at *m*/*z* 704.48745 corresponds to an ion of elemental composition C_37_H_71_O_9_NP (theoretical *m/z* = 704.487193, mass accuracy 0.37 ppm), and is thus assigned as M-H^-^ of a compound with elemental composition C_37_H_72_O_9_NP, which would correspond to a CPG. On collision induced dissociation (CID) of the *m*/*z* 704.48745 ion, two product ions were observed (Fig 2B): an ion at *m*/*z* 166.97503 with the composition C_3_H_4_O_6_P (theoretical *m/z* = 166.975098, 0.4 ppm mass accuracy), which can be assigned as deriving from the phosphoglycerate head group, and an ion at *m*/*z* 616.47012 corresponding to an ion of elemental composition of C_34_H_67_O_6_NP (theoretical *m/z* = 616.471149, 1.7 ppm mass accuracy), derived by loss from the precursor of a neutral fragment with the composition C_3_H_4_O_3_ corresponding to loss of the glycerate. We therefore propose that the lipidic compound produced by a *C*. *crescentus spt*-deficient mutant expressing *spt in trans* is CPG differing from that described by Zik et al. [14] in lacking a hydroxyl group on the lipidic portion, presumably the fatty acyl substituent.

In order to enrich for SphL I or SphL II, we separated by TLC whole lipid extracts from the *spt*-deficient mutant that expressed *spt in trans*, visualized the separated lipids and extracted SphL I or SphL II from the silica gel scraped from the plates in the areas in which the compounds corresponding to SphLs I and II migrated, before analyzing them by mass spectrometry. TLC analyses of enriched SphL I and SphL II fractions suggested that they had been separated from each other and migrated as two or one defined compounds, respectively (S1 Fig). As a control, neither of these compounds could be enriched or detected after we followed the same approach to purify SphLs I or II from an *spt*-deficient mutant of *C*. *crescentus* that harbored only an empty vector and therefore was unable to produce SphLs (S1 Fig).

**Fig 3:**
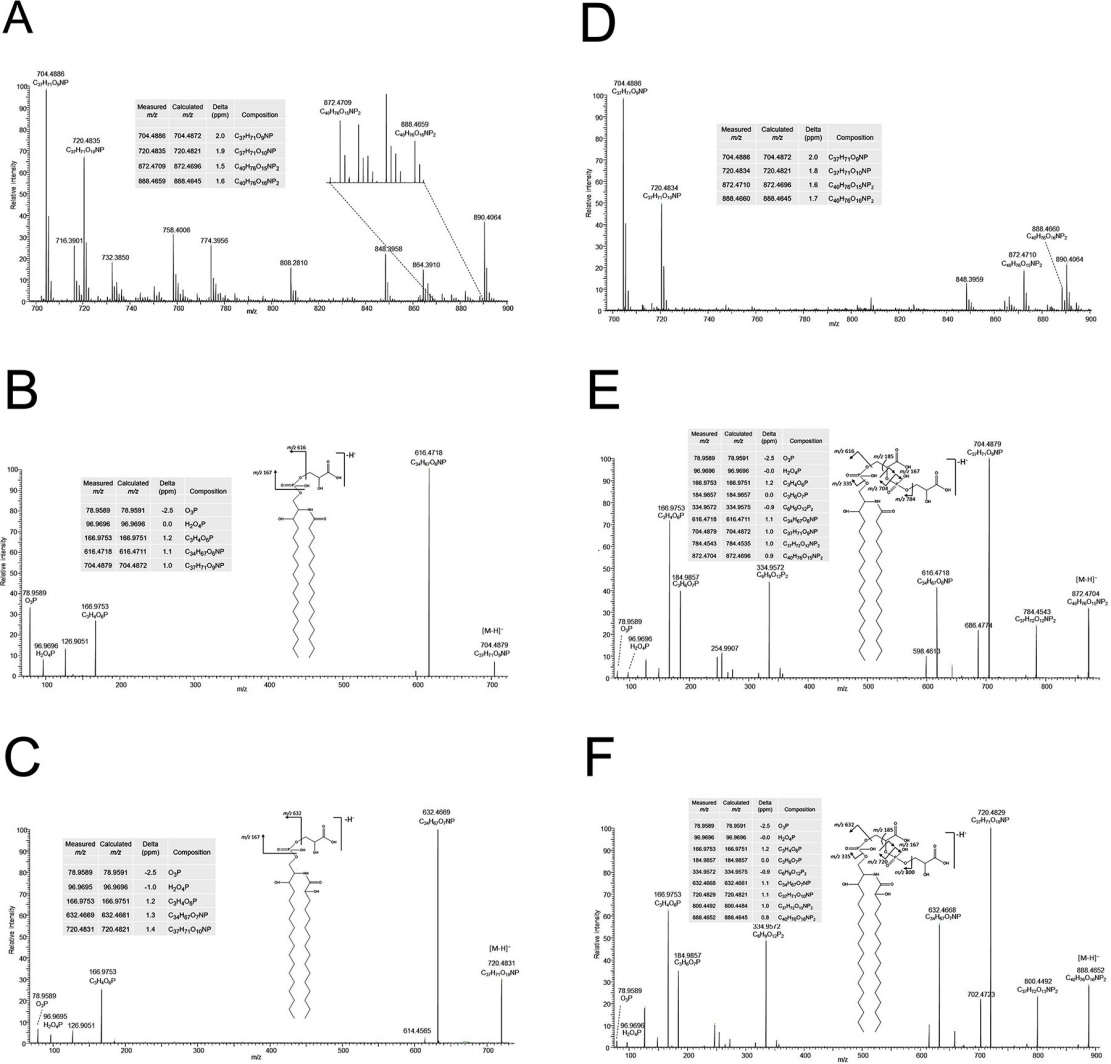
Negative ion mode ESI-Orbitrap mass spectra of lipid extracts of fractions enriched in SphL I (panel A) and SphL II (D). Negative ion mode ESI product ion spectra (obtained using HCD with product ions recorded in the Orbitrap) of the M-H^-^ precursor ions at nominal *m/z* 704 (B) and 720 (C) from the fraction enriched in SphL I, and at *m/z* 872 (E) and 888 (F) from the fraction enriched in SphL II. Note that the site of fatty acid hydroxylation indicated on the structures is presumed and cannot be assigned to C-2 of the fatty acyl group from the data presented here.

Negative mode ESI mass spectra of extracts enriched in SphL I and SphL II (Fig 3, panels A and D) bear signals consistent with the presence of two types of lipid. Intense signals at nominal *m/z* 704 and 720 are consistent with the *spt*-dependent CPG described above, and its hydroxylated counterpart respectively. The identities of these species were determined on interpretation of their high mass resolution product ion spectra (Fig 3, panels B and C respectively). In addition, less intense signals were observed in the mass spectra of both the extracts enriched in SphL I and SphL II, which correspond to di-phosphoglycerate derivatives of the two ceramide phosphoglycerates. Signals corresponding to these di-phosphoglycerates were observed at *m/z* 872.4709 (SphL I-enriched fraction) and *m/z* 872.4710 (SphL II-enriched fraction) for the unhydroxylated species, and at *m/z* 888.4659 (SphL I-enriched fraction) and 888.4660 (SphL II-enriched fraction) for their hydroxylated counterparts. The high mass resolution product ion spectra of these two components in the SphL II-enriched fraction (Fig 3, panels E and F) are clearly consistent with the presence of di-phosphoglycerate counterparts of the phosphoglycerate ceramides.

While it is not possible from these mass spectrometric data to determine absolute amounts of the different components, it is possible to consider the relative amounts of the four components in the two extracts. Comparison of the relative signal abundances of the four species in the two extracts makes it clear that the di-phosphoglycerate ceramides are enriched with respect to the mono-phoshoglycerates in the SphL II-enriched fraction compared with that of SphL I. We therefore propose that SphL I is ceramide phosphoglycerate (CPG) and that SphL II corresponds to ceramide di-phosphoglycerate (CPG2).

### Identification of genes required for phosphosphingolipid biosynthesis in *C*. *crescentus*

**Fig 4:**
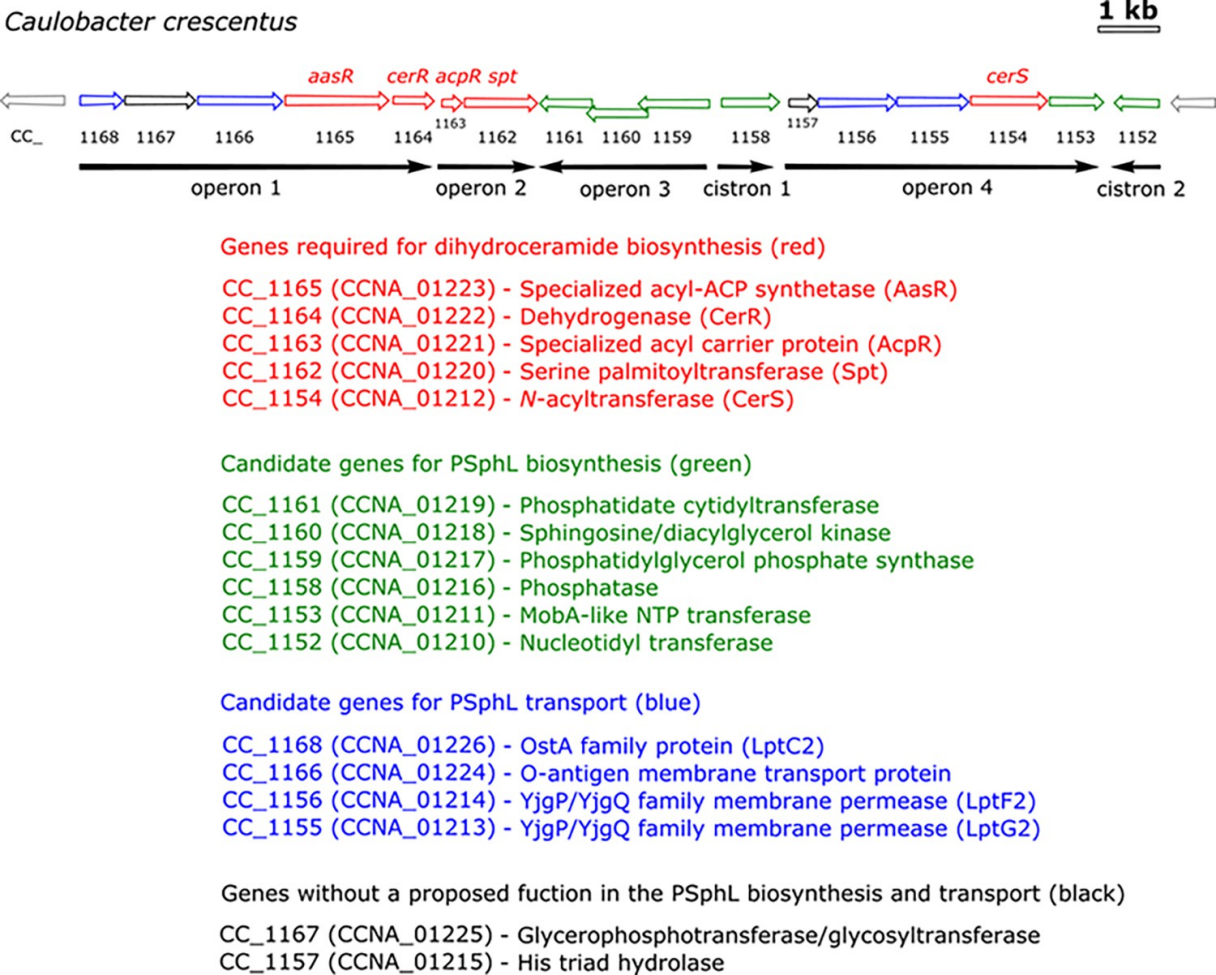
Genomic region with genes of *C*. *crescentus* involved in the biosynthesis and transport of SphLs. The 17 genes (CC_1168—CC_1152) of this region are organized into 4 operons and 2 cistrons and include genes required for dihydroceramide synthesis (CC_1165—CC_1162, and CC_1154) [9], potential genes required to convert ceramide to complex PSphLs (CC_1161—CC_1158, CC_1153, and CC_1152), genes (CC_1168, CC_1156, CC_1155) that code for second versions of LptC, LptF, LptG (i.e. LptC2, LptF2, LptG2) as well as the gene CC_1166 coding for a potential flippase and which probably participate in the selective transport of PSphLs from the IM to the OM, and structural genes probably unrelated to PSphL biosynthesis and transport (CC_1167 and CC_1157). This figure was modified from Olea-Ozuna et al. [9].

In our previous work [9], we highlighted four operons and two cistrons that are required for high fitness of *C*. *crescentus* [22] and that might be involved in SphL biosynthesis and transport (Fig 4). Specifically, CC_1165, CC_1164, CC_1163, CC_1162, and CC_1154 were shown to be required for the formation of dihydroceramide, the lipidic anchor of SphL [9]. Notably, other genes in that genomic region of *C*. *crescentus* were not required for dihydroceramide synthesis [9]. However, many mutants affected in genes from this neighborhood show remarkably high cofitness indices with a CC_1162 (*spt*)-deficient mutant [9] (S1 Table). High cofitness indices (values of 1 or slightly less) between mutants indicate that they display similarly altered phenotypes when compared with the wild type, and this suggests that the defective genes might be required for a common specific biochemical pathway [18]. The *spt*-deficient mutant shows moderate cofitness indices (in parentheses) with mutants in CC_1168 (0.52) CC_1167 (0.57), CC_1160 (0.67), CC_1159 (0.60), and CC_1158 (0.60) (S1 Table). Another group of genes related by cofitness of their mutants is associated with the predicted 2-phosphoglycerate transferase CC_1159 [14] (with cofitness indices in parentheses) with mutants in CC_1168 (0.78), CC_1167 (0.54), CC_1161 (0.79), CC_1160 (0.75), CC_1158 (0.72), CC_1156 (0.60), CC_1152 (0.74), and CC_1385 (0.53) (S1 Table).

In order to identify candidate genes that might be involved in the conversion of ceramide to PSphLs, we analyzed the genomic region comprising CC_1168—CC_1152 (S1 Text) because many of those genes were linked to ceramide-producing genes through the cofitness results of their respective mutants (S1 Table). As a result of these analyses [including predictions of protein transmembrane helices (S2 Fig)], we suggest that homologs that encode transport proteins (CC_1168, CC_1166, CC_1156, CC_1155) (Fig 4) might contribute to forming a complex transport system that moves PSphLs from their site of synthesis in the IM to their final destination in the outer layer of the OM in an analogous way to that in which LPS is transported. However, we did not investigate PSphL transport in more detail in this work. Instead, we focused on studying genes potentially coding for biosynthetic enzymes (CC_1161, CC_1160, CC_1159, CC_1158, CC_1153, CC_1152) (Fig 4) that participate in PSphL biosynthesis.

**Fig 5:**
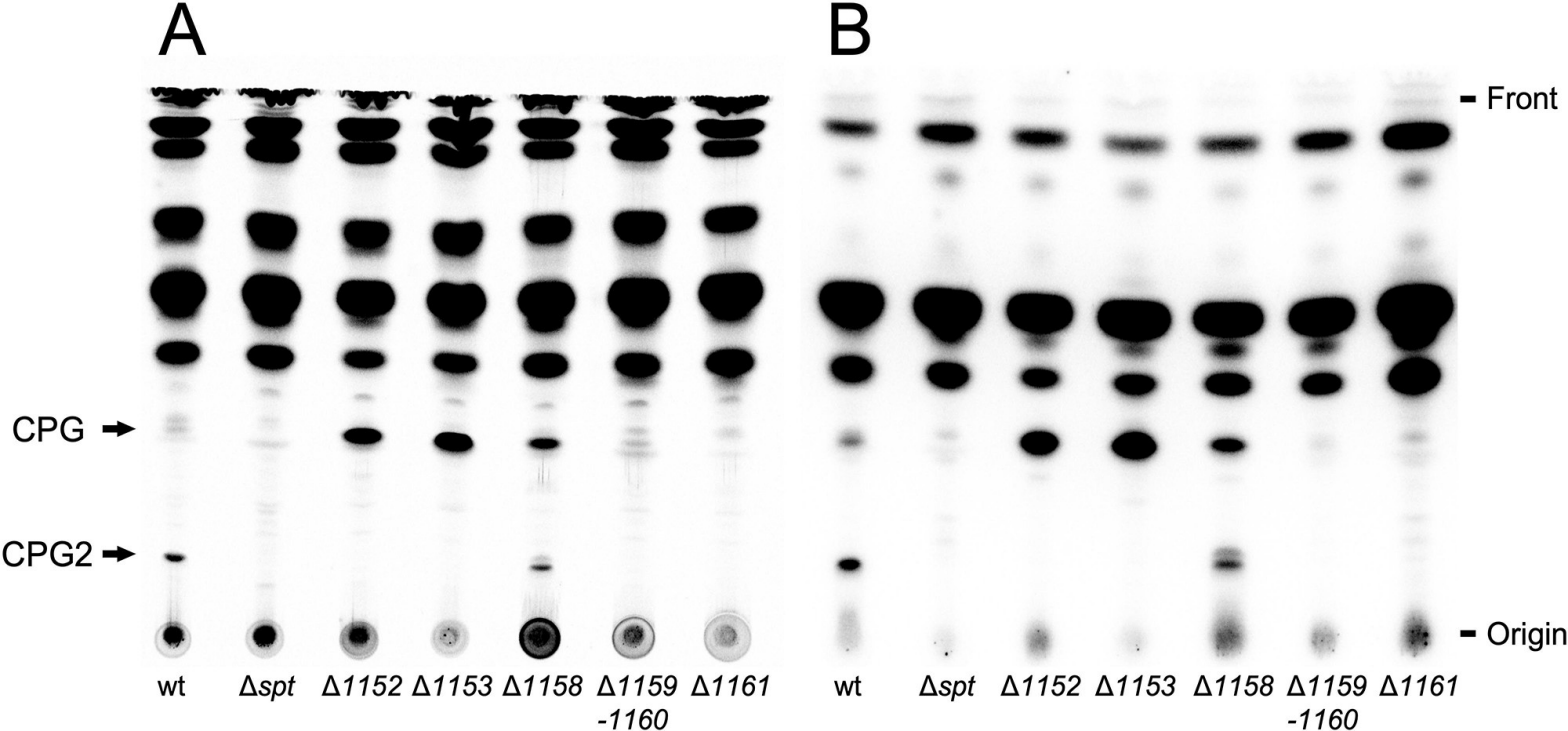
Profile of CPG and CPG2 in mutants deficient in genes required for their formation. Different strains of *C*. *crescentus* [wild type strain (wt), *spt*-deficient mutant (Δ*spt*), mutant SPG14 deficient in CC_1152 (Δ*1152*), mutant SPG15 deficient in CC_1153 (Δ*1153*), mutant SPG11 deficient in CC_1158 (Δ*1158*), mutant SPG09 deficient in CC_1159/CC_1160 (Δ*1159*/*1160*), and mutant SPG18 deficient in CC_1161 (Δ*1161*)] were cultured in complex medium in the presence of ^14^C-acetate (A) or ^32^P-phosphate (B). After harvesting cells, lipids were extracted, lipid samples were separated by TLC in chloroform/methanol/acetic acid/water (8:3:2:1) and developed chromatograms were analyzed by phosphorimaging. Arrows indicate PSphLs (CPG and CPG2) formed by *C*. *crescentus*.

Spt is required for the formation of ceramide, GSphLs [9,13], and PSphLs (Fig 1). Separation of ^14^C-acetate-labeled lipids from *C*. *crescentus* mutants deficient in CC_1161 or CC_1159/CC_1160 did not detect CPG or CPG2 (Fig 5A). In contrast, mutants deficient in CC_1153 or CC_1152 produced more intense spots for CPG than the wild type but did not produce detectable amounts of CPG2 (Fig 5A). A mutant deficient in CC_1158 produced slightly less intense CPG2 spots and slightly more intense CPG spots than the wild type (Fig 5A). When the same strains were radiolabeled with ^32^P-phosphate, results obtained with TLC-separated lipid samples support the previous statements that mutants deficient in CC_1161, CC_1159/CC_1160, CC_1153, or CC_1152 do not form CPG2 (Fig 5B), that mutants deficient in CC_1153, or CC_1152 displayed elevated levels of CPG, whereas mutants deficient in CC_1161 or CC_1159/CC_1160 were unable to produce CPG. Again, the mutant deficient in CC_1158 produced slightly less intense CPG2 spots and slightly more intense CPG spots than the wild type (Fig 5B). The accumulation of CPG in mutants deficient in CC_1152, CC_1153, or CC_1158 suggests that these three genes participate in the consumption of CPG and its conversion to CPG2.

Previously we had noted that in a mutant deficient in CC_1159/CC_1160 ceramide levels were much increased when compared to the wild type [9] and ceramide accumulation in this mutant is now observed again (S3 Fig). Also, in the mutant deficient in CC_1153 much increased ceramide levels were detected, whereas the mutant deficient in CC_1161 displays moderately increased ceramide levels. In mutants deficient in CC_1152 or CC_1158 ceramide levels are below the levels encountered in the wild type (S3 Fig). The accumulation of ceramide in mutants deficient in CC_1153, CC_1161, or CC_1159/CC_1160 suggests that these genes contribute to the consumption of ceramide and its conversion to CPG and CPG2.

When performing complementation experiments, the intact gene(s) were expressed *in trans* in the respective mutants or the mutants harbored an empty vector. Expression of CC_1152 in a CC_1152-deficient mutant shows reduced levels of CPG and increased levels of CPG2 when compared to the CC_1152-deficient mutant harboring the empty vector (S4A Fig). Also, expression of CC_1153 in a CC_1153-deficient mutant shows reduced levels of CPG and increased levels of CPG2 when compared to the CC_1153-deficient mutant harboring the empty vector. These results suggest that CC_1152 and CC_1153 contribute to the reduction of CPG and are required for the formation of CPG2.

Expression of CC_1158 in a CC_1158-deficient mutant shows reduced levels of CPG and increased levels of CPG2 when compared to the CC_1158-deficient mutant harboring the empty vector (S4A Fig). Similarly to the mutant deficient in CC_1159/CC_1160 (Fig 5), the CC_1161 mutant harboring an empty vector did not produce detectable levels of CPG or CPG2. However, when CC_1160 was expressed in the CC_1159/CC_1160-deficient mutant, CPG was formed which was not the case when CC_1159 was expressed instead (S4A Fig). When expressing CC_1159 and CC_1160 in the CC_1159/CC_1160-deficient mutant, formation of CPG and CPG2 was restored (S4A Fig). Finally, expression of CC_1161 in a CC_1161-deficient mutant restored formation of CPG and CPG2 which was not the case when the CC_1161-deficient mutant harbored an empty vector (S4A Fig). When the same strains were radiolabeled with ^33^P-phosphate, mutants in CC_1158, CC_1153, or CC_1152 harboring the empty vector showed increased levels of CPG when compared to the analogous mutants expressing the intact genes *in trans* (S4B Fig). Expression of CC_1159 in mutants CC_1159/1160 displayed the same lipid profile as this mutant harboring the empty vector (S4B Fig). Expression of CC_1159 and CC_1160 was required for the formation of CPG2 (S4B Fig). Notably, expression of CC_1160 in mutant CC_1159/1160 led to increased CPG formation (S4B Fig). This result indicates that CC_1160, but not CC_1159, is required for CPG formation. When the mutant deficient in CC_1161 was complemented with CC_1161 *in trans*, CPG and CPG2 were formed, which was not the case for the mutant harboring the empty vector (S4B Fig).

**Fig 6:**
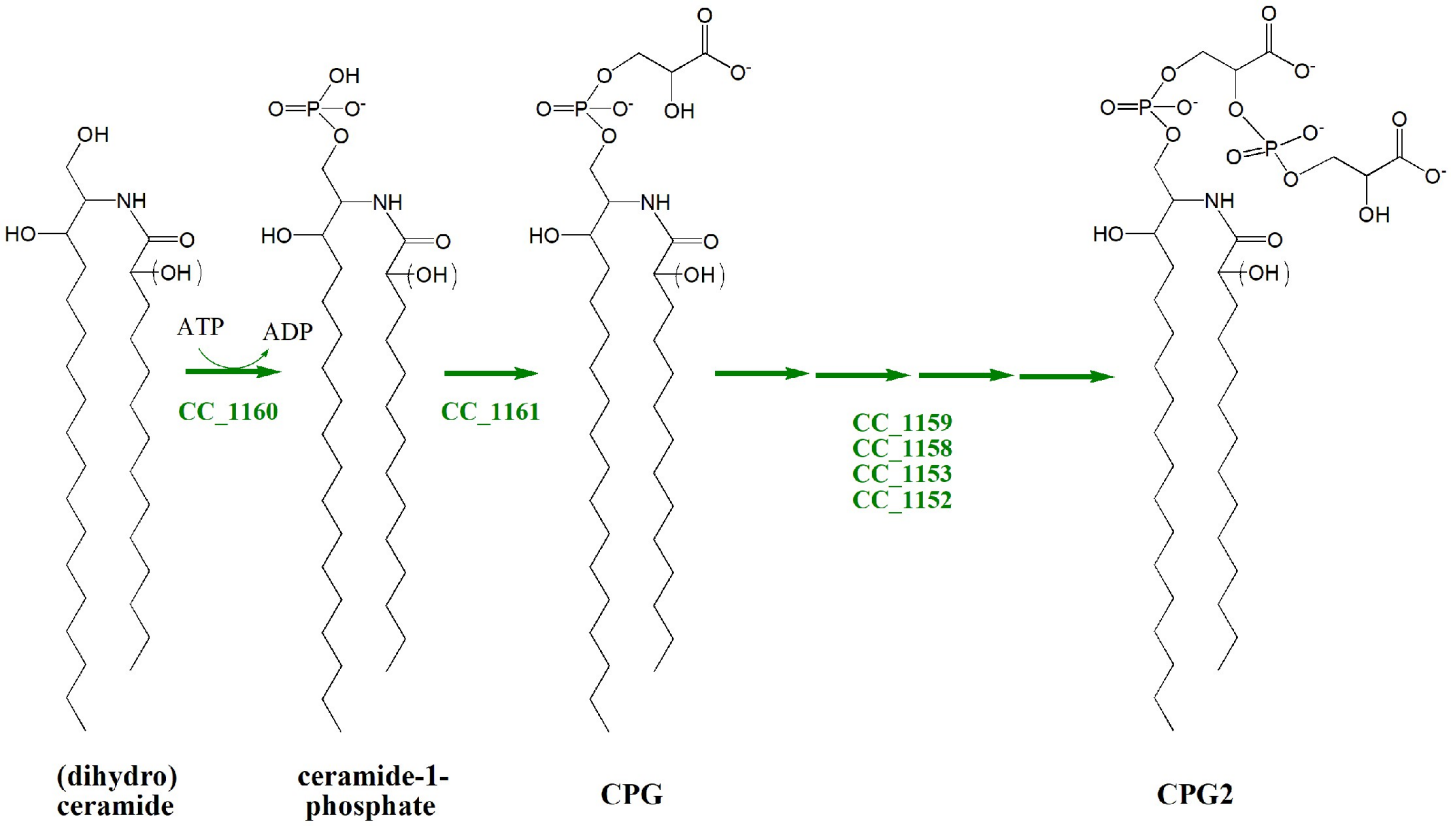
Model for genes involved in biosynthesis of PSphLs CPG and CPG2 in *C*. *crescentus*. TLC lipid analyses of the wild type strain and the different mutants required in PSphL formation support the model that genes CC_1160 and CC_1161 are required for CPG (SphL I) formation, while genes CC_1159, CC_1158, CC_1153, and CC_1152 are involved in CPG2 (SphL II) formation. All four lipid structures shown may carry or not a hydroxy group at an undefined position of their fatty acyl residue.

Therefore, TLC studies of lipids of the wild type strain, different mutant strains, and mutant strains expressing intact genes *in trans* in *C*. *crescentus* suggest that the CC_1161 and CC_1160 genes are required for efficient CPG formation, while the CC_1159, CC_1158, CC_1153, and CC_1152 genes are necessary for the efficient formation of CPG2 (Fig 6). Recently, an enzyme assay for CC_1160 has been developed [23], clarifying that CC_1160 is a ceramide kinase which converts ceramide to ceramide-1-phosphate in the initial step of PSphL biosynthesis (Fig 6). CC_1161 must be involved in a subsequent step during CPG formation, but we presently do not know in which order the remaining gene products participate in CPG2 formation (Fig 6). As CC_1152 and CC_1153 are essential for CPG2 formation and as CC_1158 contributes to it, the scheme we propose for CPG2 synthesis is more complex (Fig 6) than that suggested previously by Zik *et al*. [14]. However, future work needs to clarify the enzymatic functions of CC_1161, CC_1159, CC_1158, CC_1153, and CC_1152 in order to be able to define the precise biosynthesis pathway for CPG and CPG2.

### Putative CPG and CPG2 biosynthesis pathway is conserved in some other proteobacteria

**Fig 7:**
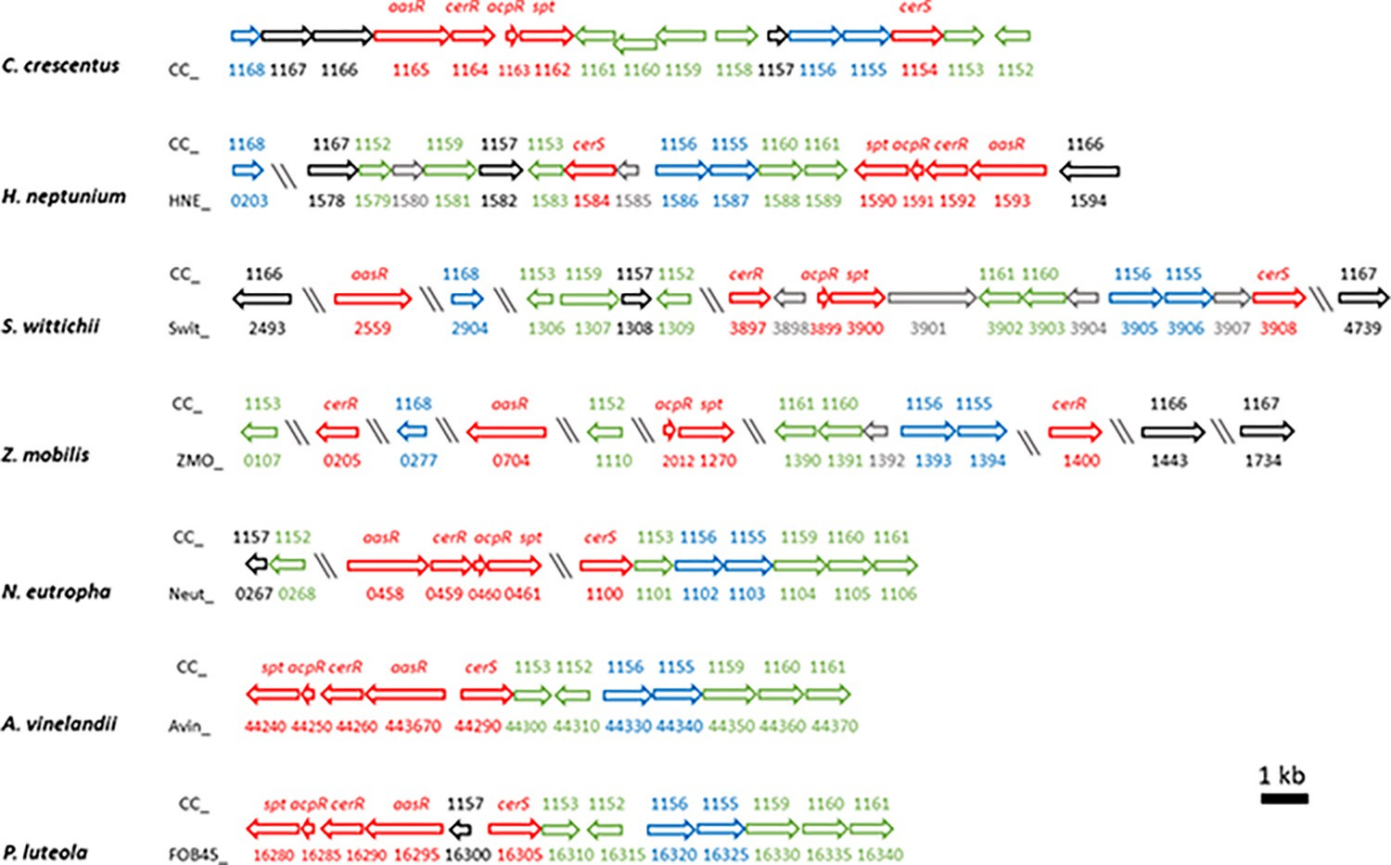
Genomic organization of genes/operons for phosphosphingolipid biosynthesis and transport in *Rhodobacteria*. Operons containing genes involved in the biosynthesis and transport of phosphospingolipids from *Caulobacter crescentus*, *Hyphomonas neptunium*, *Sphingomonas wittichii*, *Zymomonas mobilis*, *Nitrosomonas eutropha*, *Azotobacter vinelandii*, and *Pseudomonas luteola* are shown. Five genes, the orthologs of *aasR*, *cerR*, *acpR*, *spt*, and *cerS*, are required for ceramide biosynthesis (red arrows). Six genes (CC_1160, CC_1161, CC_1159, CC_1158, CC_1153, CC_1152) participate in the biosynthesis of the PSphL CPG2 (green arrows). Four genes may be involved in transport of SphLs from the IM to the OM (blue arrows), and two genes (CC_1157 and CC_1167) are probably unrelated to PSphL biosynthesis and transport (black arrows). For each organism, assigned gene names are indicated below the arrows, while the orthologs from *C*. *crescentus* are spelled out above the arrows, respectively. Large genomic distances are indicated by inclined double bars. Accession numbers for the genes mentioned above and relevant criteria for assignment are listed in S2 Table and its legend.

Structural genes for ceramide biosynthesis (*aasR*, *cerR*, *acpR*, *spt*, *cerS*) are conserved within the *Rhodobacteria*, the group which comprises α-, β-, and γ-proteobacteria [8]. Using gene products of CC_1168-CC_1152 for BLAST P searches in bacterial genomes, we detected orthologs in *Hyphomonas neptunium* (S2A Table), *Sphingomonas wittichii* (S2B Table), *Zymomonas mobilis* (S2C Table), *Nitrosomonas eutropha* (S2D Table), *Azotobacter vinelandii* (S2E Table), and *Pseudomonas luteola* (S2F Table), not only for the five ceramide biosynthesis proteins, but also for other gene products of the 17 genes-involving cluster (CC_1168-CC_1152) (Fig 7 and S2 Table).

*H*. *neptunium* is a close relative of *C*. *crescentus*, lacks a CC_1158 ortholog, but has good orthologs for the other 16 genes (Fig 7 and S2A Table). As CC_1158 was not essential for CPG2 formation in *C*. *crescentus*, we would expect that *H*. *neptunium* can still make CPG and CPG2.

Similarly, *S*. *wittichii* lacks the CC_1158 ortholog and has largely good orthologs for the other 16 genes (Fig 7 and S2B Table). However, although these orthologs are distributed over 6 distant regions in the genome, *S*. *wittichii* should be able to produce CPG and CPG2.

*Z*. *mobilis* lacks CC_1159 and CC_1157 in addition to CC_1158 and has only moderate orthologs of CC_1153 and CC_1152 (Fig 7 and S2C Table). Therefore, *Z*. *mobilis* possesses the structural genes for making CPG but not for the synthesis of CPG2. The genes for the other remaining 14 orthologs of *Z*. *mobilis* are distributed over 10 distant regions in the genome. Therefore, in *Sphingomonadales*, such as *S*. *wittichii* or *Z*. *mobilis*, we can observe that the genes for ceramide or CPG2 synthesis and for potential SphL transport are scattered over the genomes.

Genomes of the β-proteobacterium *N*. *eutropha* as well as of the γ-proteobacteria *A*. *vinelandii* and *P*. *luteola* lack CC_1168, CC_1167, CC1166 orthologs in addition to the CC_1158 ortholog. However, for at least 13 genes of the 17 genes-involving cluster (CC_1168-CC_1152) good orthologs exist in *N*. *eutropha* (S2D Table), *A*. *vinelandii* (S2E Table), and *P*. *luteola* (S2F Table) and all 3 bacterial strains should be able to form CPG and CPG2. Whereas the orthologs are distributed over 3 regions in the *N*. *eutropha* genome, in the γ-proteobacteria *A*. *vinelandii* and *P*. *luteola* they are contiguous and located to one genomic region (Fig 7). Therefore, during evolution these γ-proteobacteria might have received their ceramide/CPG2 biosynthesis/transport cluster by horizontal gene transfer from a gene cluster-containing α-proteobacterium, such as *C*. *crescentus*.

Even in *C*. *crescentus*, not necessarily all genes required for CPG2 synthesis are located in the same genomic region as the *spt* gene. A recent report suggests that the CC_0202 (CCNA_00202) gene, predicted to encode a DesA family fatty acid desaturase/hydroxylase, is responsible for the hydroxylation of the amide-linked fatty acyl residue of SphLs [2]. Interestingly, a CC_0202-deficient mutant of *C*. *crescentus* lacking this hydroxyl group, becomes resistant to polymyxin E (colistin) [2], suggesting that this hydroxylation is required to ensure that CPG2 can act as a functional polymyxin receptor. In addition, hydroxylations at the 2 or 3 position of fatty acyl residues of complex membrane lipids, occupying the outer leaflet of the OM, are not uncommon and they are expected to participate in an extensive hydrogen-bonding network with neighboring molecules leading to a more stable membrane [5].

### Resistance and sensitivity of *C*. *crescentus* mutants affected in PSphL biosynthesis towards polymyxin B

**Fig 8:**
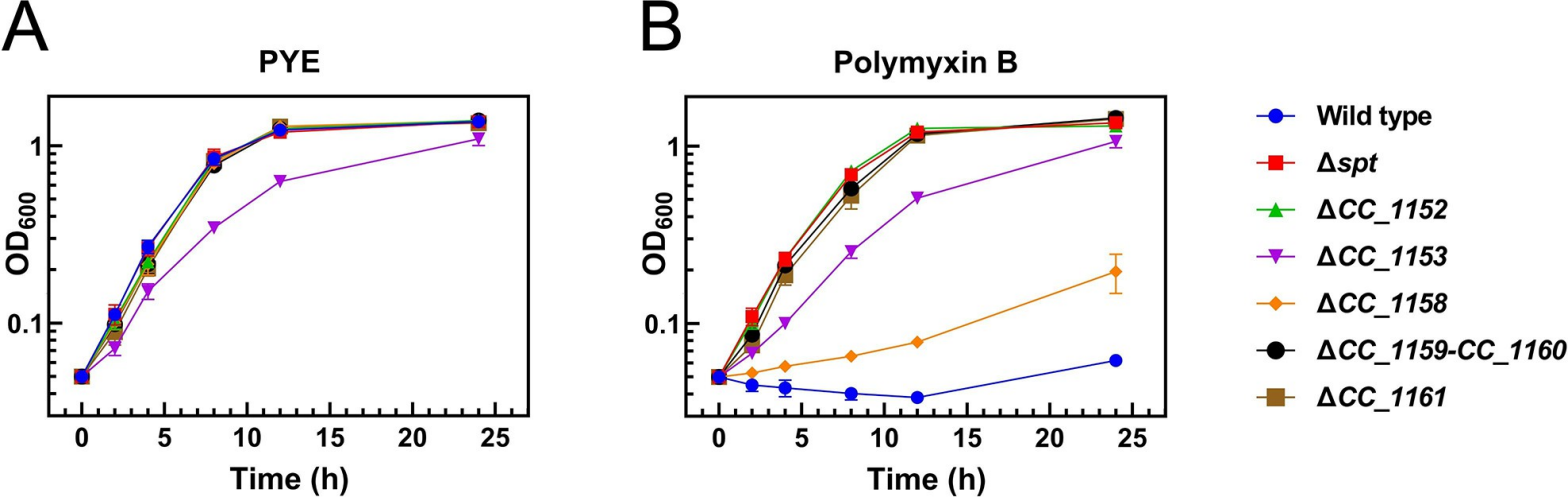
Genes for PSphL formation are required for sensitivity of *C*. *crescentus* to polymyxin B. Growth (OD_600_) of different strains of *C*. *crescentus* was determined at 30°C on complex medium (A) or complex medium in the presence of polymyxin B (10 μg/ml) (B). Data and bars represent the average and standard errors obtained from at least three independent experiments.

Polymyxins are cationic antimicrobial peptides which may serve as last-resort antibiotics with potent activity against multi-drug resistant pathogens. Polymyxin B seems to interact initially with the 4´-phosphate group of the lipid A portion of the LPS-containing OM [24], replacing bivalent cations and thereby weakening interactions between LPS molecules. Then polymyxin B is thought to cross the OM in a self-promoting uptake mechanism and after interacting with the IM provokes lysis and cell death [25]. *C*. *crescentus* is devoid of phosphate residues on the lipid A part of its LPS [26] and therefore LPS might not be the main target for polymyxin B in this bacterium. In our previous work [9] we showed that CC_1165, CC_1164, CC_1163, CC_1162, and CC_1154, the genes required for ceramide formation, were needed to confer polymyxin B sensitivity on *C*. *crescentus*. In the presence of polymyxin B, mutants deficient in CC_1159/CC_1160, CC_1161, or CC_1152 grew much more rapidly than the wild type and similarly to the CC_1162 (*spt*)-deficient mutant (Fig 8). Expression of CC_1159, CC_1160, or CC_1159/CC_1160 in the CC_1159/CC_1160-deficient mutant reveals that only the combined expression of CC_1159 and CC_1160 restored polymyxin B sensitivity (S5D Fig). Expression of CC_1161 in the CC_1161-deficient mutant (S5E Fig), or expression of CC_1152 in a CC_1152-deficient mutant (S5A Fig), restored polymyxin B sensitivity, whereas the presence of the empty vectors did not. A mutant deficient in CC_1158 grew more rapidly than the wild type, but more slowly than the CC_1162 (*spt*)-deficient mutant in the presence of polymyxin B (Fig 8), displaying an intermediate phenotype. The expression of intact CC_1158 in the CC_1158-deficient mutant eliminated growth of *C*. *crescentus* in polymyxin-containing medium (S5C Fig). Although CC_1153 was thought to be an essential gene [22], we were able to generate a deletion mutant deficient in CC_1153. However, a CC_1153-deficient mutant grows much more slowly than the wild type in complex PYE medium (Fig 8A) and this growth behavior is not altered upon cultivation in the presence of polymyxin B (Fig 8B); therefore, we conclude this mutant is also polymyxin-resistant. Expression of intact CC_1153 in the CC_1153-deficient mutant eliminated growth in polymyxin-containing medium (S5B Fig), suggesting that CC_1153 contributes to the formation of a PSphL that confers polymyxin sensitivity on *C*. *crescentus*. Therefore, in addition to the five ceramide biosynthesis genes described previously [2,8,9], the six genes participating in the conversion of ceramide to the PSphL CPG2 are also required for generating polymyxin sensitivity. LPS of *C*. *crescentus* lacks phosphate groups and therefore is probably not a target for polymyxin B. In contrast, the negatively charged PSphL CPG2, or a derivative of it, might constitute the main target molecule for polymyxin in *C*. *crescentus*.

### Phosphosphingolipids of *C*. *crescentus* are required for resistance towards deoxycholate

**Fig 9:**
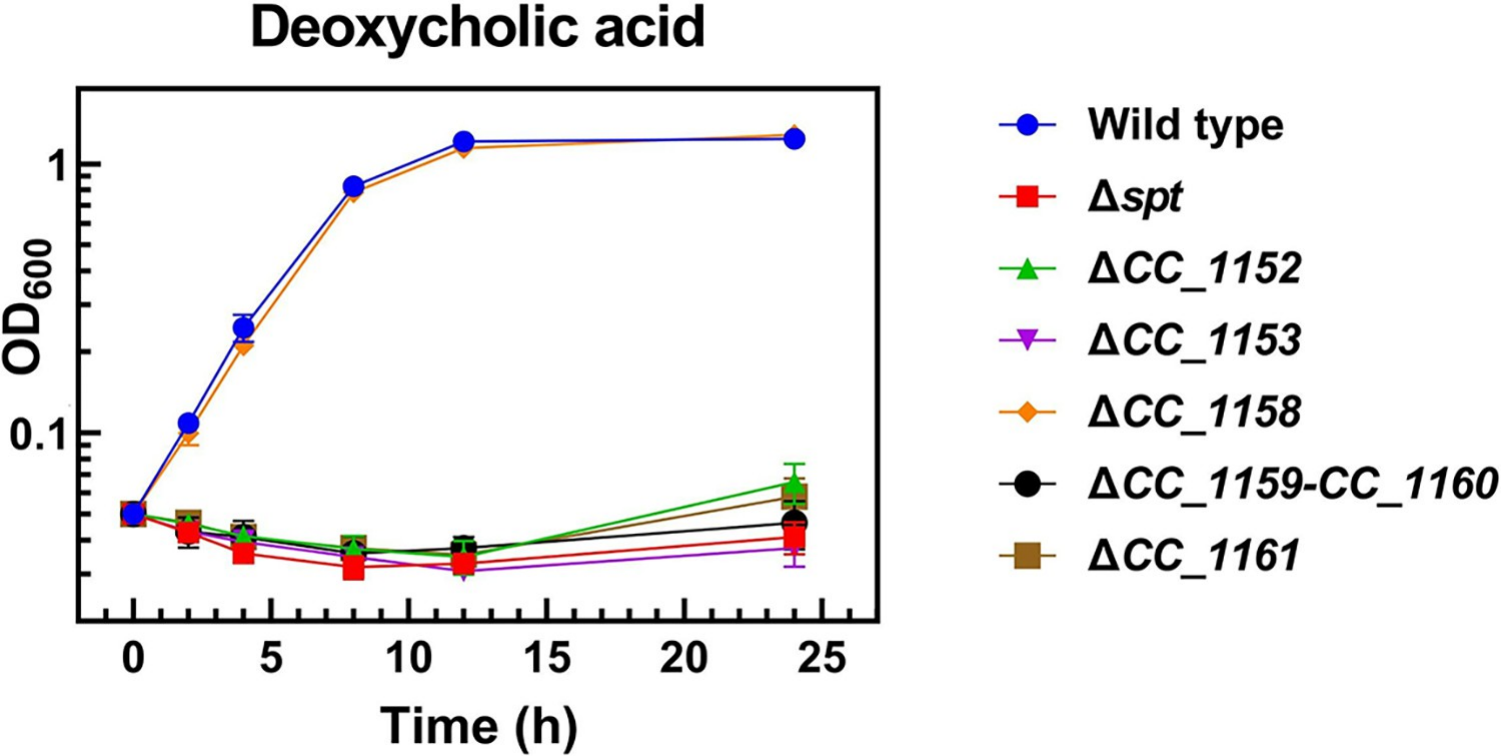
Genes for PSphL formation are required for resistance of *C*. *crescentus* to deoxycholate. Growth (OD_600_) of different strains of *C*. *crescentus* [wild type strain (Wild type), *spt*-deficient mutant (Δ*spt*), mutant SPG14 deficient in CC_1152 (Δ*1152*), mutant SPG15 deficient in CC_1153 (Δ*1153*), mutant SPG11 deficient in CC_1158 (*Δ1158*), mutant SPG09 deficient in CC_1159/CC_1160 (Δ*1159*–*1160*), and mutant SPG18 deficient in CC_1161 (Δ*1161*)] was determined at 30°C on complex medium in the presence of deoxycholate (1 mg/ml). Data and bars represent the average and standard errors obtained from at least three independent experiments.

Bile acids such as deoxycholate act as biological detergents and are able to emulsify and solubilize many cell membranes. However, OMs of Gram-negative bacteria contain LPS and sometimes SphLs in the outer leaflets of their OM, which show high capacities for bridging interactions by divalent cations and for lateral hydrogen bond interactions [5], thereby leading to a very rigid membrane which is more resistant to treatment with detergents. The formation of ceramide in *C*. *crescentus* is required for resistance towards the detergent deoxycholate [9]. We have now studied whether the formation of PSphLs is also required for this resistance. Whereas the wild type strain and the CC_1158-deficient mutant grew well in deoxycholate-containing medium, mutants deficient in CC_1152, CC_1153, CC_1159/CC_1160, and CC_1161, like the *spt*-deficient mutant, did not grow in the presence of deoxycholate (Fig 9). However, when mutants in CC_1152, CC_1153, CC_1159/CC_1160, and CC_1161 were complemented with the respective intact genes *in trans* they were able to grow in deoxycholate-containing medium, which was not the case if they harbored an empty vector instead (S6 Fig). From these data, we conclude that the formation of PSphL CPG2 in *C*. *crescentus* is required for conferring resistance towards deoxycholate. Therefore, in addition to the five ceramide biosynthesis genes described previously [2,8,9], five genes participating in the conversion of ceramide to CPG2 are also required for generating resistance towards the detergent deoxycholate and therefore for membrane stability. The fact that the mutant deficient in CC_1158 grows like the wild type in the presence of deoxycholate suggests that in this mutant enough CPG2 or a derivative of it is still formed to provide deoxycholate resistance.

### Phosphosphingolipids of *C*. *crescentus* are required for virulence on *Galleria mellonella* larvae

In recent years, larvae of the greater wax moth *Galleria mellonella* have become an important animal model for studying virulence. Pathogens injected into *G*. *mellonella* provoke cellular and humoral responses by the larvae [27]. Notable humoral immune responses include the formation of antimicrobial peptides and the activation of phenol oxidase leading to melanin formation and melanization of the larvae when counteracting an immune challenge due to invasive microbes [27]. Recent studies have shown that distinct *Caulobacter* species are virulent in the *G*. *mellonella* infection model [21]. In order to analyze whether bacterial SphLs might be responsible for this virulence, we tested the effect of various *C*. *crescentus* strains when injected into *G*. *mellonella* larvae. Over a period of five days, we distinguished healthy larvae from melanized and dead larvae.

When setting up healthspan assays, we initially inoculated PYE medium, nonvirulent *E*. *coli*, or virulent *Burkholderia cenocepacia* [28] strains into *G*. *mellonella* larvae. Injection of PYE medium or of *E*. *coli* did not decrease the percentage of healthy larvae in a significant way over a 5 day period (S7 Fig). In contrast, injection of *B*. *cenocepacia* led to a steady decline in healthy larvae over the first 3 days (S7 Fig); by then all larvae were either melanized or dead.

**Fig 10:**
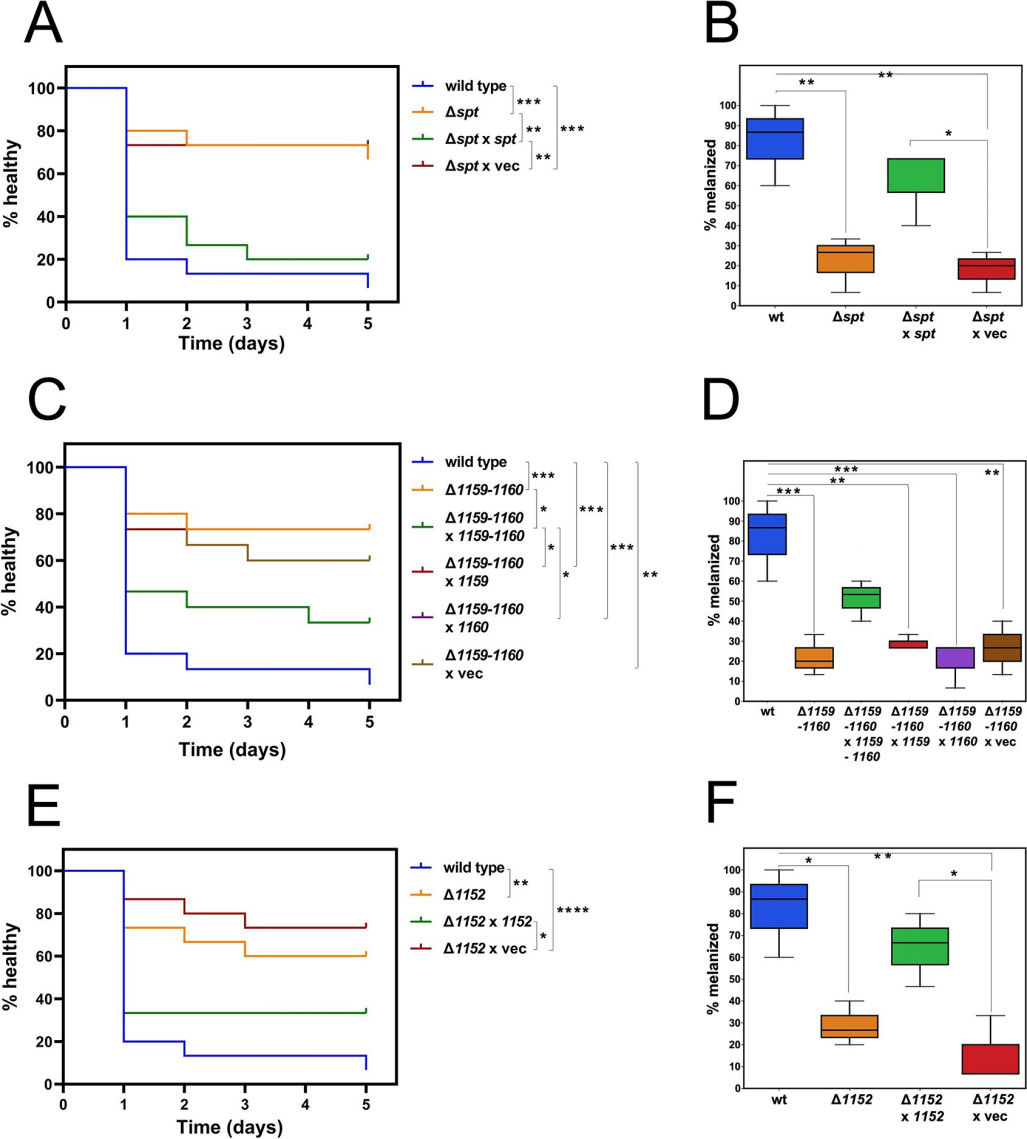
PSphL formation is required for virulence of *C*. *crescentus* in *G*. *mellonella* larvae. Healthspan of *G*. *mellonella* larvae was determined with Kaplan-Meier survival analysis up to 5 days after inoculation with distinct *C*. *crescentus* strains (left column). Melanized *G*. *mellonella* larvae 24 h after inoculation of the larvae with distinct *C*. *crescentus* strains (right column). Inoculation of *G*. *mellonella* larvae was performed with 10^5^ CFU of wild type (wt) *C*. *crescentus* strain, *spt*-deficient mutant (Δ*spt*), *spt*-deficient mutant expressing intact *spt in trans* (Δ*spt* x *spt*), *spt-*deficient mutant harboring the empty vector (Δ*spt* x vec) (A, B), CC_1159/CC_1160-deficient mutant (Δ*1159–1160*), CC_1159/CC_1160-deficient mutant expressing intact CC_1159/CC_1160 *in trans* (Δ*1159–1160* x *1159–1160*), CC_1159/CC_1160-deficient mutant expressing intact CC_1159 *in trans* (Δ*1159–1160* x *1159*), CC_1159/CC_1160-deficient mutant expressing intact CC_1160 *in trans* (Δ*1159–1160* x *1160*), CC_1159/CC_1160-deficient mutant harboring the empty vector (Δ*1159–1160* x vec) (C, D), CC_1152-deficient mutant (Δ*1152*), CC_1152-deficient mutant expressing intact CC_1152 *in trans* (Δ*1152* x *1152*), or CC_1152-deficient mutant harboring the empty vector (Δ*1152* x vec) (E, F), respectively. Survival curves are a representative cohort (n = 15) of at least three biological replicates (Mantel-Cox test for statistics, *P<0.05, **P<0.01, ***P<0.001, ****P<0.0001).

Whereas healthy larvae declined rapidly when inoculated with *C*. *crescentus* wild type, this was not the case when a *spt*-deficient mutant of *C*. *crescentus* was applied instead (Fig 10A and 10B). An *spt*-deficient mutant complemented with the intact *spt* gene *in trans* caused rapid decrease of healthy larvae and therefore restored *C*. *crescentus* virulence in *G*. *mellonella*, whereas the *spt*-deficient mutant harboring an empty vector did not (Fig 10A). Therefore, as Spt is required for the formation of any SphL, bacterial SphLs are required for *C*. *crescentus* virulence. Remarkably, most of the melanization provoked by *C*. *crescentus* in *G*. *mellonella* larvae occurred within the first 24 h after injection (Fig 10A and 10B), but even after 5 days a few larvae had survived and were not melanized. Statistical analyses show that the percentage of melanized larvae after 24 h is significantly larger when inoculated with the *C*. *crescentus* wild type than with the *spt*-deficient mutant (Fig 10B). Also, inoculation of larvae with a *spt*-deficient mutant complemented with the intact *spt* gene *in trans* caused a significantly higher percentage of melanized larvae when compared with larvae that had been inoculated with a *spt*-deficient mutant harboring an empty vector (Fig 10B).

Similarly, whereas healthy larvae declined rapidly when inoculated with *C*. *crescentus* wild type, this was not the case when a CC_1159/CC_1160-deficient (Fig 10C and 10D) or a CC_1152-deficient (Fig 10E and 10F) mutant of *C*. *crescentus* was injected instead. A CC_1159/CC_1160-deficient mutant complemented with the intact CC_1159/CC_1160 genes (Fig 10C and 10D) or a CC_1152-deficient mutant complemented with the CC_1152 gene (Fig 10E and 10F) caused a rapid decrease in healthy larvae and therefore restored virulence in *G*. *mellonella* whereas the CC_1159/CC_1160-deficient mutant (Fig 10C and 10D) or the CC_1152-deficient mutant (Fig 10E and 10F) harboring an empty vector *in trans*, respectively, did not. Expression of CC_1159 or CC_1160 alone in the CC_1159/CC_1160-deficient mutant did not restore virulence of *C*. *crescentus* in *G*. *mellonella* larvae (Fig 10C and 10D) either. Statistical analyses show that the percentage of melanized larvae after 24 h is significantly larger when inoculated with the *C*. *crescentus* wild type than with the CC_1159/CC_1160-deficient mutant (Fig 10D) or with the CC_1152-deficient mutant (Fig 10F). In addition, inoculation of larvae with a CC_1159/CC_1160-deficient mutant complemented with the intact CC_1159/CC_1160 genes *in trans* (Fig 10D) or with a CC-1152-deficient mutant complemented with the CC_1152 gene (Fig 10F) caused a significantly higher percentage of melanized larvae when compared with larvae that had been inoculated with a CC_1159/CC_1160-deficient (Fig 10D) or a CC_1152-deficient (Fig 10F) mutant harboring an empty vector, respectively, or vectors expressing only the CC_1159 or CC_1160 gene (Fig 10D). As CC_1160 is required for the formation of any PSphL (Figs 5 and S4), bacterial PSphLs are required for *C*. *crescentus* virulence and as CC_1152 is required for the formation of CPG2, this bacterial PSphL or derivatives of it are required for *C*. *crescentus* virulence in *G*. *mellonella* larvae. Therefore, *C*. *crescentus* can be used as a surrogate system to study the role of SphLs in virulence and pathogenesis.

In previous work, Moore and Gitai [21] suggested that the virulence-provoking compound was contained in the LPS fraction. We therefore analyzed LPS fractions of *C*. *crescentus* wild type and mutants affected in ceramide/CPG2 biosynthesis genes (S8 Fig). These data show that all mutants contain the same carbohydrate-containing fractions as the wild type and that most of the mutants display similar levels of the different carbohydrate-containing fractions as the wild type (including mutants deficient in *spt*, *CC1159/CC1160*, or *CC_1152*, which were used in the *G*. *mellonella* virulence assay). All LPS fractions present in wild type were also present in all of the mutants (S8 Fig), suggesting that the absence of SphLs and specifically of CPG2 does not lead to major alterations of the LPS fractions. Therefore, this present study shows that genes required for SphL, PSphL, and specifically CPG2 formation are needed for *C*. *crescentus* virulence on larvae of the greater wax moth *G*. *mellonella* and that this virulence is not due to major alterations of LPS fractions in *C*. *crescentus*.

Mutants deficient in *spt*, CC_1159/CC1160, or CC_1152 are defective in ceramide, CPG, or CPG2 formation, respectively, and as they are sensitive to the detergent deoxycholate, their OM might be impaired when compared to wild type. We now studied growth and survival of the strains by following optical density (OD) and the number of colony forming units (CFU) per ml over time. At 30°C, growth behavior of the *spt*-deficient mutant was indistiguishable from the wild type, i.e. the OD or CFU per ml values were very similar for both strains, which held true for exponential and stationary phases of growth (S9 Fig). Also, the other mutants affected in the conversion of ceramide to CPG2 (mutants deficient in CC_1159/CC_1160 or in CC_1152) showed very similar growth behavior as wild type or the *spt*-deficient mutant (S9 Fig). Clearly, under the mentioned conditions of growth, the three mutants studied are at no disadvantage with regard to the wild type and we conclude that their OM was sufficiently intact to permit wild type-like growth and survival.

### Conclusions

The present analysis suggests that besides the five genes required for ceramide synthesis in *C*. *crescentus*, another six genes (CC_1161, CC_1160, CC_1159, CC_1158, CC_1153, CC_1152) from this genomic region participate in the formation of the PSphL CPG2, the presumptive target molecule for interaction with polymyxin, and therefore responsible for the trait of polymyxin sensitivity in *C*. *crescentus*. Ten of the eleven genes required for the biosynthesis of the PSphL CPG2 are needed for an intact bacterial cell envelope and for the formation of a cell-associated factor that provokes virulence on larvae of the greater wax moth *G*. *mellonella*. The mutant deficient in CC_1158 often shows intermediate phenotypes, which might suggest the existence of another gene that can partially substitute for CC_1158.

Although we probably know most of the genes required for the conversion of ceramide to CPG2, the precise biochemical synthesis pathway still needs to be defined. The first step for CPG2 biosynthesis consists in the conversion of ceramide to ceramide-1-phosphate by the CC_1160-encoded ceramide kinase. Based on mass spectrometric data, it had been proposed that CC_1161 attaches a glycerate residue to ceramide-1-phosphate. Although this proposal seems energetically not very favorable, we contemplate this proposal in our biosynthesis scheme (Fig 6). Remarkably, CC_1161 contains an InterPro domain that suggests it might be a CDP-diacylglycerol (CDP-DAG) synthase, an enzyme known to catalyze CDP-DAG formation from phosphatidic acid. Therefore, one might expect that CC_1161 is involved in an activation step. For example, by consuming a nucleoside triphosphate (NTP), CC_1161 might form a nucleoside diphosphate (NDP)-activated ceramide from ceramide-1-phosphate. For the condensation of glycerate or 2-phospho-glycerate to the activated ceramide derivative, another (so-far unknown) gene/enzyme might be required and CPG or 2-phospho-CPG could be formed. In our biosynthesis scheme (Fig 6), we indicate that four other gene products might be involved in order to convert CPG or 2-phospho-CPG to CPG2. CC_1153 shows similarity to an InterPro domain of 2-phospho-L-lactate guanilyltransferase CofC, a nucleotidyltransferase that participates in the biosynthesis of coenzyme F420. CC_1153 might catalyze the consumption of a NTP and convert 2-phospho-CPG to a 2-NDP-activated CPG. CC_1152 also possesses PFAM domains which suggests it might be another nucleotidyltransferase and it shows sequence similarity to the CDP-choline synthase LicD and the CDP-glycerol synthase Gtp2. We therefore suggest that CC_1152 might consume a NTP and convert 2-phospho-glycerate to a 2-NDP-activated glycerate, which might serve as one of the substrates in the CC_1159-catalyzed reaction. CC_1159 has a PFAM domain which suggests displacement of CMP from a CDP-alcohol in order to condense with a second alcohol. Therefore, CC_1159 might catalyze the condensation of 2-NDP-activated CPG with 2-NDP-glycerate generating 2-NDP-CPG2. Finally, CC_1158 has a phosphoesterase InterPro domain, is expected to cleave phosphoester bonds, thereby removing NDP and producing CPG2. Clearly, assays need to be developed for these novel enzymes that would confirm or correct the presented scenario.

The immune system of *G*. *mellonella* larvae is structurally and functionally similar to the innate immune response of mammals. Functions exerted by the PSphL CPG2 in the insect larvae might occur at least on two levels. Firstly, after infection of the larvae with *C*. *crescentus*, outer membrane vesicles carrying CPG2 might be released from the *C*. *crescentus* OM and these liberated CPG2 molecules might activate immune cells termed hemocytes in the hemolymph. Secondly, as the version of CPG2, which is hydroxylated in the amidified fatty acyl residue, seems to be required to confer polymyxin sensitivity to *C*. *crescentus*, CPG2 is expected to act as OM receptor for the antimicrobial peptide polymyxin. The melanization observed in *G*. *mellonella* after treatment with CPG2-producing *C*. *crescentus* strains is part of the humoral response exerted by hemocytes. Another aspect of the hemocyte-mediated immune response includes the release of antimicrobial defense peptides and it is tempting to speculate that some of them might be specifically recognized by CPG2 in an initial step leading finally to bacterial cell lysis.

## Materials and methods

### Bacterial strains, plasmids, and growth conditions

The bacterial strains and plasmids used and their relevant characteristics are shown in Table 1. The construction of *C*. *crescentus* mutants deficient in putative SphL biosynthesis genes is described in S3 Table. Strains of *C*. *crescentus*, *Escherichia coli* MG1655, or *Burkholderia cenocepacia* J2315 were grown in complex peptone yeast extract (PYE) medium [29] at 30°C on a gyratory shaker at 250 rpm. For growth experiments, strains were first grown on PYE plates. Then, cells were resuspended at cell densities of 5 x 10^7^ cells/ml in liquid PYE medium and grown for 20 h during such a first growth cycle. During a second subcultivation in PYE medium, again inoculating with 5 x 10^7^ cells/ml, growth of *C*. *crescentus* wild type and of SphL-deficient mutants was followed by determining OD_600_. For studying bacterial growth in the presence or absence of polymyxin B, deoxycholate, cultures with 15 ml medium in 125 ml Erlenmeyer flasks were employed.

**Table 1:** Bacterial strains and plasmids.

Strain or plasmid	Relevant characteristics	Reference
*Caulobacter crescentus*		
*C*. *crescentus* CB15N	Wild type used throughout this study, synchronizable derivative of CB15	[31]
CB15N derivatives		
DAGS01	*CC_1162*::*deletion*	[9]
SPG09	*CC_1159/1160*::*deletion*	[9]
SPG11	*CC_1158*::*deletion*	[9]
SPG14	*CC_1152*::*deletion*	This study
SPG15	*CC_1153*::*deletion*	This study
SPG18	*CC_1161*::*deletion*	This study
*Escherichia coli*		
DH5α	*supE*44, Δ*lac*U169, ϕ80 *lacZ*ΔM15, *hsdR*17, *recA*1, *endA*1, *gyrA*96, *thi*-1, *relA*1, host for cloning	[32]
TOP10	F^-^, *mcrA* Δ(*mrr-hsdRMS-mcrBC*) ϕ80 *lacZ*ΔM15, Δ*lacX*74, *recA1*, *ara*Δ139, Δ(*ara-leu*)7697 *galU galK*, *rpsL* (Sm^R^), *endA1*, *nupG*, host for cloning	Invitrogen
S17-1	*thi*, *pro*, *recA*, *hsdR*, *hsdM+*, RP4Tc::Mu, Km::Tn*7*;Tp^R^, Sm^R^, Sp^R^	[33]
MG1655	F^-^, λ^-^, *ilv*G^-^, *rfb*-50, *rph*-1	[34]
*Burkholderia cenocepacia* J2315		[35]
Plasmids		
pET17b	Expression vector, Cb^R^	Novagen
pNPTS138	Suicide vector, Km^R^	MRK Alley
pRXMCS-2	Expression vector, Km^R^, low copy number	[36]
pBXMCS-2	Expression vector, Km^R^, high copy number	[36]
pRJ08	pRXMCS-2 carrying *CC_1162*	[9]
pRJ21	pBXMCS-2 carrying *CC_1152*	This study
pRJ22	pBXMCS-2 carrying *CC_1153*	This study
pRJ23	pBXMCS-2 carrying *CC_1158*	This study
pRJ24	pBXMCS-2 carrying *CC_1159*	This study
pRJ25	pBXMCS-2 carrying *CC_1160*	This study
pRJ26	pBXMCS-2 carrying *CC_1159/CC_1160*	This study
pRJ27	pBXMCS-2 carrying *CC_1161*	This study

^a^Tp^R^, Km^R^, Sp^R^, Sm^R^, Cb^R^: trimethoprim, kanamycin, spectinomycin, streptomycin, carbenicillin, resistance respectively.

Other *E*. *coli* strains were cultured on Luria-Bertani (LB) medium [30] at 37°C. Antibiotics were added to media in the following concentrations (μg/ml) when required: kanamycin 5 (25 in solid media), polymyxin B 10, in the case of *C*. *crescentus*, and kanamycin 50, carbenicillin 50, in the case of *E*. *coli*.

Plasmids pBXMCS-2, pNPTS138, and their derivatives were moved into *C*. *crescentus* by conjugation. In the conjugation, aliquots of exponentially growing cultures of a donor strain of *E*. *coli* S17-1, harboring the plasmid of interest (200 μl), and of the receiver strain *C*. *crescentus* CB15N (1 ml) were mixed. The mixed cell suspension was centrifuged and washed three times with ice-cold 10% glycerol, resuspended in 100 μl of PYE medium and applied in drops onto PYE agar. After most of the liquid had evaporated, the cell mixture was incubated at 30°C for 16 h and potential transconjugants were selected on PYE agar containing nalidixic acid (25 μg/ml) and kanamycin (25 μg/ml). Expression of proteins in *C*. *crescentus* from pRXMCS-2 or pBXMCS-2 derivatives was achieved by adding xylose to a final concentration of 10 mM to cultures at an OD_600_ = 0.05.

### DNA manipulations and analyses

Recombinant DNA techniques were performed according to standard protocols [37]. Commercial sequencing of amplified genes by Eurofins Medigenomix (Martinsried, Germany) corroborated the correct DNA sequences. The DNA regions containing *cc_1168-cc_1152* were analyzed using the NCBI (National Center for Biotechnology Information) BLAST network server [38].

### Construction of expression plasmids

Using PCR and a pair of specific oligonucleotides (S4 Table) genes or combinations of genes encoding potential structural genes for SphL biosynthesis were amplified from *C*. *crescentus* genomic DNA. Suitable restriction sites for cloning of the genes were introduced by PCR with oligonucleotides. After restriction with the respective enzymes, the PCR-amplified DNA fragments were cloned into a pET17b or a pBXMCS-2 or recloned into a pBXMCS-2 vector as detailed in S4 Table.

### *In vivo* labeling of bacterial strains with [^14^C] acetate, [^32^P] phosphate or [^33^P] phosphate

The lipid composition of bacterial strains was determined after labeling with [1-^14^C] acetate (60 mCi/mmol; Perkin Elmer), [^32^P] phosphate (1 Ci/mmol; Perkin Elmer), or [^33^P] phosphate (1 Ci/mmol; American Radiolabeled Chemicals, Inc.). Cultures (1 ml) of wild type or mutant strains were inoculated from precultures grown in the same medium in order to obtain an initial cell density of 2 x 10^8^ cells/ml. After the addition of 1 μCi [1-^14^C] acetate, 2.5 μCi [^32^P] phosphate, or of 2.5 μCi [^33^P] phosphate to each culture, they were incubated for 16 h. At the end of the respective incubation periods, cells were harvested by centrifugation, and resuspended in 100 μl of water. Lipids were extracted according to the method of Bligh and Dyer [39] and the chloroform phase was separated by one-dimensional TLC on high performance TLC aluminum sheets (silica gel 60; Merck Poole, United Kingdom) and developed with chloroform/methanol/ammonium hydroxide (40:10:1; v/v) or chloroform/methanol/acetic acid/water (8:3:2:1; v/v) as the mobile phase. Radioactive lipids were visualized by phosphorimaging using a Typhoon FLA 9500 and quantification was performed with Image Quant TL (Amersham Biosciences).

### Mass spectrometric analysis

For mass spectrometric analyses, usually bacterial cultures comprising 100 ml of medium were grown, lipids were extracted as described above [39] and studied directly. In the case of enriched SphL I or SphL II fractions, the *spt*-deficient mutant expressing *spt in trans* or harboring an empty vector, was cultivated in 2 l of medium to an OD_600_ of 1.2, when cells were harvested, lipids extracted, and separated by TLC on silica gel-containing plates. After separation with chloroform/methanol/acetic acid/water (8:3:2:1; v/v) as the mobile phase, lipids were visualized using iodine vapor and silica gel fractions containing SphL I or SphL II were scraped from the plates and lipids were extracted from the silica gels. Aliquots of the enriched SphL I or SphL II fractions were reanalyzed by TLC and did not contain major other lipidic compounds that could be detected by staining with iodine vapor [40] or with 8-anilo-1-naphthalenesulfonic acid (ANS) reagent [41].

High resolution mass spectra were acquired with a solariX XR FT-ICR mass spectrometer equipped with a 9.4 T superconducting magnet (Bruker Daltonics) and an Orbitrap Fusion Tribrid mass spectrometer (Thermo Scientific). Spectra were acquired in negative ion mode using ESI; lipid samples were redissolved in dichloromethane:methanol (1:1, v:v) and further diluted in methanol; solutions were introduced into the ion source by syringe infusion with a flow rate of 2–3 μL min^−1^.

SolariX: ESI conditions were spray voltage 3500 V, nebuliser gas pressure 2 bar, drying gas flow 4 L min^−1^; temperature 160°C. External calibration was performed on sodium formate clusters with a 10 μg mL^−1^ solution in 50% propan-2-ol. Mass spectra were also internally calibrated on fatty acid peaks using linear calibration. This procedure results in a mass accuracy better than 0.5 ppm for the mass spectrometric signals reported (2 ppm for product ion signals). The spectra were acquired with 1 M data points over the *m/z* range 100–2000 (transient of 0.367 s) resulting in a resolving power of 37000 at *m/z* 700. CID was performed by isolation (width *m/z* 2.0) of the precursor ions in the quadrupole and then storage in the hexapole collision cell with an excitation voltage of 23 V. The ion accumulation time in the ion source was set to 0.1 s (0.5 s for product ion spectra). A total of 8 scans were added for each mass spectrum. Spectra visualization and formula calculation was performed with DataAnalysis 5.0 (Bruker Daltonics).

Orbitrap Fusion: HESI source settings were spray voltage 3300 V, sheath gas 2.5 Arb, aux gas 0.5 Arb, ion transfer tube temperature 275°C. Spectra were acquired with 120000 resolution at *m/z* 200 (64000 at *m/z* 700), scan range *m/z* 200–1000 for mass spectra and auto for product ion spectra. Easy-IC was used for internal calibration. For product ion spectra, isolation (width *m/z* 1.6) was carried out in the quadrupole followed by HCD with excitation voltage 25–32 V. A total of approximately 1 minute of acquired data were averaged to produce each spectrum. Spectra visualization and formula calculation was performed with Xcalibur (version 4.0; Thermo Scientific).

### *Galleria mellonella* healthspan assay

All *G*. *mellonella* larvae were obtained from PETMMAL, S.A. DE C.V. (Cuautitlán Izcalli, México) and were kept in an incubator at 30°C. The larvae were used for health tests within three days of receiving the package. For virulence experiments, *C*. *crescentus* strains were resuspended to an OD_600_ of 0.05 and in PYE medium and cultivated until reaching an OD_600_ of 0.125 (10^7^ CFU/ml). Cells were washed three times with PYE medium and resuspended in the original volume. A 31G (gauge) insulin syringe was used to inoculate 10 μl of bacterial suspension, which corresponds to 10^5^ CFU. Virulence was tested using 3 cohorts of 15 *G*. *mellonella* larvae placed in Petri dishes without diet and incubated at 30°C. Melanization and mortality were assessed every 24 h for 5 days after injection. Healthy larvae were neither melanized nor dead.

### Raw data for bacterial growth curves and assays with *G*. *mellonella*

Raw data for bacterial growth curves and *G*. *mellonella* larval assays are provided in S1 Data.

## Supporting information

S1 Text Bioinformatic analyses of CC_1168—CC_1152 candidate genes for sphingolipid biosynthesis and transport. (DOCX)

S1 Table Cofitness of mutants affected in genes putatively involved in PSphL biosynthesis and transport in *C*. *crescentus*. Cofitness data were taken from Price *et al*. [18]. Mutants in CC_1157 do not show high cofitness values with other mutants of the cluster. For CC_1155 and CC_1153 no data have been reported. CC_1165, CC_1164, CC_1163, CC_1162, or CC_1154 are indicated as *aasR*, *cerR*, *acpR*, *spt*, or *cerS*, respectively.
(DOCX)

S2 Table Orthologs of sphingolipids biosynthesis and transport proteins from *C*. *crescentus* in selected *Rhodobacteria* (α-, β-, and γ-proteobacteria), i.e. in *H*. *neptunium* (S2A Table), *S*. *wittichii* (S2B Table), *Z*. *mobilis* (S2C Table), *N*. *eutropha* (S2D Table), *A*. *vinelandii* (S2E Table), and *P*. *luteola* (S2F Table). (DOCX)

S3 Table Construction of *C*. *crescentus* knock-out mutants in potential sphingolipid biosynthesis genes. (DOCX)

S4 Table Oligonucleotides used for amplification of different sphingolipid biosynthesis genes. Sites for recognition by restriction enzymes are underlined.
(DOCX)

S5 Table Construction of different expression plasmids. For details see Materials and Methods in main text.
(DOCX)

S1 Fig Fractions in SphL I and SphL II after purification by TLC. Lipid extracts from large cultures (2 l) of the *spt*-deficient mutant harboring the *spt* gene *in trans* (Δ*spt* x *spt*) or of the *spt*-deficient mutant harboring the empty vector pRXMCS-2 (Δ*spt* x vec) of *C*. *crescentus* were obtained, separated by preparative TLC, and visualized on exposure to iodine vapor. From areas that contained SphL I or II, silica gel was scraped, lipids were extracted, and aliquots were reanalyzed by TLC in order to assess their purity. As visualized after iodine or ANS staining, fractions enriched in SphL I (panel A) or SphL II (panel B) were obtained from the SphL-producing Δ*spt* x *spt* strain while no analogous lipids were observed following the same extraction procedures carried out on the Δ*spt* x vec strain of *C*. *crescentus*.
(TIF)

S2 Fig Prediction of transmembrane helices for CC_1168, CC_1166, CC_1160, CC_1159, CC_1156, and CC_1155 using the bioinformatic programs TMHMM 2.0 (left) and Phobius (right). (PDF)

S3 Fig Dihydroceramide profiles of mutants deficient in genes required for PSphL formation. Different *C*. *crescentus* strains [wild-type strain (wt), *spt*-deficient mutant (Δ*spt*), mutant SPG14 deficient in CC_1152 (Δ*1152*), mutant SPG15 deficient in CC_1153 (Δ*1153*), mutant SPG11 deficient in CC_1158 (Δ*1158*), mutant SPG09 deficient in CC_1159–1160 (Δ*1159*–*1160*), and mutant SPG18 deficient in CC_1161 (Δ*1161*)] were cultured in complex medium in the presence of ^14^C-acetate. After harvesting cells, lipids were extracted, separated by TLC in chloroform/methanol/ammonium hydroxide (40:10:1) and developed chromatograms were analyzed by phosphorimaging. Arrows indicate dihydroceramides formed by *C*. *crescentus*.
(TIF)

S4 Fig Complementation of *C*. *crescentus* mutants altered in the formation of PSphL (CPG and CPG2) by their native genes *in trans*. Mutants of *C*. *crescentus* deficient in CC_1152, CC_1153, CC_1158, CC_1159–1160, or CC_1161 carrying the respective intact gene in the xylose-inducible plasmid pBXMCS-2 (Δ*1152* x *1152*, Δ*1153* x *1153*, Δ*1158* x *1158*, Δ*1159*–*1160* x *1159*, Δ*1159*–*1160* x *1160*, Δ*1159*–*1160* x *1159*–*1160*, Δ*1161* x *1161*) or carrying the empty pBXMCS-2 vector (vec) *in trans* were radiolabeled with ^14^C-acetate (A) or ^33^P-phosphate (B) for 16 h. At the end of the labeling period, cells were harvested, lipids were extracted, separated by TLC in chloroform/methanol/acetic acid/water (8:3:2:1) and developed chromatograms were subjected to autoradiography. Arrows indicate PSphLs (CPG and CPG2) formed by *C*. *crescentus*.
(TIF)

S5 Fig Complementation of *C*. *crescentus* mutants altered in the PSphL formation by their native genes *in trans* restores sensitivity to polymyxin B. Growth (OD_600_) of *C*. *crescentus* mutants deficient in CC_1152 harboring the CC_1152-expressing plasmid (Δ*1152* x *1152*) or the empty vector pBXMCS-2 (Δ*1152* x vec) (A), mutants deficient in CC_1153 harboring the CC_1153-expressing plasmid (Δ*1153* x *1153*) or the empty vector pBXMCS-2 (Δ*1153* x vec) (B), mutants deficient in CC_1158 harboring the CC_1158-expressing plasmid (Δ*1158* x *1158*) or the empty vector pBXMCS-2 (Δ*1158* x vec) (C), mutants deficient in CC_1159–1160 harboring the CC_1159-1160-expressing plasmid (Δ*1159–1160* x *1159–1160*), the CC_1159-expressing plasmid (Δ*1159–1160* x *1159*), the CC_1160-expressing plasmid (Δ*1159–1160* x *1160*), or the empty vector pBXMCS-2 (Δ*1159–1160* x vec) (D), and mutants deficient in CC_1161 harboring the CC_1161-expressing plasmid (Δ*1161* x *1161*) or the empty vector pBXMCS-2 (Δ*1161* x vec) (E). Data and bars represent the average and standard errors obtained from at least three independent experiments. Note that growth curves for strains Δ*1159–1160* x *1159*, Δ*1159–1160* x *1160*, and Δ*1159–1160* x vec overlap in (D).
(TIF)

S6 Fig Genes for PSphL formation are required for resistance of *C*. *crescentus* to deoxycholate. Growth (OD_600_) of different strains of *C*. *crescentus* [mutant SPG14 deficient in CC_1152 expressing intact CC_1152 *in trans* (Δ*1152 x 1152*) or mutant harboring the empty vector (Δ*1152 x* vec)(A), mutant SPG15 deficient in CC_1153 expressing intact CC_1153 *in trans* (Δ*1153 x 1153*) or mutant harboring the empty vector (Δ*1153 x* vec)(B), mutant SPG09 deficient in CC_1159/CC_1160 expressing intact CC_1159/CC_1160 *in trans* (Δ*1159*–*1160 x 1159/1160*) or mutant harboring the empty vector (Δ*1159–1160 x* vec)(C), and mutant SPG18 deficient in CC_1161 expressing intact CC_1161 *in trans* (Δ*1161 x 1161*) or mutant harboring the empty vector (Δ*1161 x* vec)(D)] was determined at 30°C on complex medium in the presence of deoxycholate (1 mg/ml). Data and bars represent the average and standard errors obtained from at least three independent experiments.
(TIF)

S7 Fig Healthspan assays of *G*. *mellonella* larvae was determined with Kaplan-Meier survival analysis up to 5 days after inoculation with PYE medium, *E*. *coli* MG1655 (10^5^ CFU), or *B*. *cenocepacia* J2315 (10^5^ CFU). Survival curves are a representative cohort (n = 15) of at least three biological replicates (Mantel-Cox test for statistics, ***P<0.001).
(TIF)

S8 Fig Lipopolysaccharide profiles of *C*. *crescentus* wild type and mutants affected in sphingolipid biosynthesis genes. Hot aqueous-phenol LPS extractions were performed exactly as described [14] from 1 ml of exponentially growing cultures (OD_600_ = 0.8) of *C*. *crescentus* wild type (wt) and mutants affected in ceramide formation (Δ*spt*, Δ*1154*, Δ*1164*, Δ*1165*) or in further conversion of ceramide to CPG2 (Δ*1161*, Δ*1159/1160*, Δ*1158*, Δ*1153*, Δ*1152*). Samples, corresponding to 1/40 of the original culture, were analyzed by gel electrophoresis (16.5%) in a Mighty Small II equipment (Hoefer). Carbohydrates were stained using Pro Q Emerald 300 Lipopolysaccharide Gel Stain Kit (Molecular Probes; P20495) per manufacturer´s instructions.
(TIF)

S9 Fig Lack of ceramide or CPG2 synthesis does not affect growth or survival of *C*. *crescentus* at normal growth temperature. Growth and survival of wild type, *spt*-deficient mutant DAGS01 (Δ*spt*), CC_1159/CC_1160-deficient mutant SPG9 (Δ*1159/1160*), or CC_1152-deficient mutant SPG14 (Δ*1152*) was determined at 30°C on complex medium. Growth of the *C*. *crescentus* strains was followed by measuring OD_600_ (filled symbols) whereas survival was quantified by determining CFU per ml (open symbols). Data and bars represent the average and standard errors obtained from at least three independent experiments.
(TIF)

S1 Data Raw data for bacterial growth curves and larval assays. Raw data for generating Figs 8–10 and S5, S6, and S9 Figs are given.
(XLSX)
