# Supplementary material for: Genes required for phosphosphingolipid formation in Caulobacter crescentus contribute to bacterial virulence
Source: PLoS Pathog. 2024 Aug 2;20(8):e1012401. doi: 10.1371/journal.ppat.1012401 (PMC11324152; doi:10.1371/journal.ppat.1012401)
Supplement: S1 Text — (DOCX) [file ppat.1012401.s001.docx]

**S1 Text. Bioinformatic analyses of CC_1168 - CC_1152 candidate genes for sphingolipid biosynthesis and transport**

In order to identify candidate genes that might be involved in the conversion of ceramide to PSphLs, we analyzed the genomic region comprising CC_1168 - CC_1152 because many of those genes were linked to ceramide-producing genes through the cofitness results of their respective mutants.

CC_1168 displays the PFAM domain PF06835 which belongs to the LptC family. Following extraction of LPS from the IM, LptC was shown to participate in the transport of LPS to the OM of Gram-negative bacteria [1]. In *C. crescentus*, CC_1168 might be needed for PSphL transport from the IM to the OM. When predicting transmembrane protein topology with a hidden Markov model (TMHMM) [2] or Phobius [3], CC_1168 has a possible transmembrane helix close to its N-terminus (S2 Fig). Fitness browser suggests that CC_1168 mutants display cofitness with mutants deficient in CC_1167, CC_1161, CC_1160, CC_1159, CC_1158, CC_1156, and CC_1152 (S1 Table).

CC_1167 has the InterPro domain IPR007554, suggesting that it might encode a CDP-glycerol glycerophosphotransferase or glycosyltransferase. A ceramide-UTP-glucuronosyltransferase-deficient mutant of *Zymomonas mobilis* accumulates ceramide-phospho-glycerol [4]. There are good homologs (ZMO1734; E = 4e-83 and Swit_4739; E = 8e-99) of CC_1167 encoded by the *Z. mobilis* and *Sphingomonas* *wittichii* genomes, respectively. But presently their exact function is unknown.

CC_1166 has the InterPro domain IPR002797, suggesting that it might encode an oligosaccharide transport membrane protein (flippase) involved in synthesis of the O-antigen of LPS. According to TMHMM and Phobius, CC_1166 has nine transmembrane helices (S2 Fig). The MsbA protein from *E. coli* (ECK0905; b0914) has a close ortholog (CC_0305; CCNA_00307) encoded in the *C. crescentus* genome (97% coverage, E value 4e-114, 35% identity, 56% similarity) and MsbA is the flippase that transports lipid A-core from the inner leaflet to the outer leaflet of the IM [5]. In contrast, the CC_1166 protein is quite different from MsbA and the fact that mutants deficient in CC_1166 share moderate cofitness with mutants deficient in CC1156 or CC_1152 (S1 Table) strengthen the argument that CC_1166 might be a flippase that transports PSphLs from the inner leaflet of the IM to its outer leaflet.

CC_1165 encodes the specialized acyl-ACP synthetase AasR which specifically acylates the special acyl carrier protein AcpR [6].

CC_1164 is predicted to encode an NADPH-dependent epimerase/dehydrogenase and is probably responsible for the reduction of the 3-oxo group in 3-oxo-sphinganine. However, recently it was suggested that CC_1164 would encode a dehydrogenase, also termed CerR [7], that reduces *N*-acylated 3-oxo-sphinganine (3-oxo-dihydroceramide) and that *N*-acylation of 3-oxo-sphinganine by CC_1154, also termed bCerS [7], would precede the reduction step [7].

CC_1163 is the specialized acyl carrier protein AcpR, required for efficient SphL biosynthesis in *Rhodobacteria* [6].

CC_1162 encodes an Spt that preferentially uses palmitoyl-AcpR as acyl donor during 3-oxo-sphinganine synthesis [6].

There are homologs of CC_1161 in α-, β-, γ-, and δ-proteobacteria and they contain the InterPro domain IPR015222 of “mitochondrial cytidylyltransferase phosphatides” (Tam41/Mmp7) (CDP-DAG synthase) which catalyzes CDP-DAG formation from phosphatidic acid. Fitness browser suggests that CC_1161-deficient mutants display cofitness with mutants deficient in CC_1168, CC_1160, CC_1159, CC_1158, CC_1156, CC_1152, CC_1385 (predicted fructose-1,6-bisphosphatase) (S1 Table), and CC_0001 (putative pyruvate water, dikinase/phospenolpyruvate synthetase). It is remarkable that CC_1385 as well as CC_0001 seem to encode enzymes participating in gluconeogenesis. Based on mass spectrometric data, Zik *et al*. [8] propose that CC_1161 attaches a glycerate residue to ceramide-1-phosphate.

CC_1160 was annotated as sphingosine/diacylglycerol (DAG) kinase. TMHMM predicts a transmembrane helix in CC_1160 with an N-terminal cytoplasmic and a C-terminal periplasmic domain (S2 Fig), while Phobius predicts two transmembrane helices (S2 Fig). Fitness browser suggests that CC_1160 mutants display cofitness with mutants deficient in CC_1168, CC_1167, CC_1161, CC_1159, CC_1158, and CC_1156 (S1 Table). Recent experimental work indicates that CC_1160 is a novel ceramide kinase that can phosphorylate ceramide as well as DAG [8, 9].

CC_1159 has the PFAM domain PF01066 which suggests displacement of CMP from a CDP-alcohol in order to condense with a second alcohol. Both TMHMM and Phobius suggests an N-terminal cytoplasmic domain and three transmembrane helices for CC_1159 (S2 Fig). Based on mass spectrometric data, Zik *et al*. [8] propose that CC_1159 is a 2-phosphoglycerate transferase.

CC_1158 has a phosphoesterase InterPro domain IPF004843 similar to that of calcineurin type ApaH. These phosphoesterases are specific for 3´, 5´-cAMP. Fitness browser suggests cofitness of mutants affected in CC_1168, CC_1167, CC_1161, CC_1160, CC_1159, CC_1156, or CC_1152 with a mutant deficient in CC_1158 (S1 Table).

CC_1157 has a histidine triad motif (HIT) consisting of HXHXHXX, where X is a hydrophobic amino acid. Proteins with HIT domains form a superfamily of nucleotide hydrolases and transferases which act on the α-phosphate of ribonucleotides.

CC_1156 contains a PFAM domain PF03739 (LptG/LptF) and codes for an integral membrane protein (TMHMM and Phobius predicts 6 transmembrane helices) (S2 Fig) which might participate in the export of LPS or PSphLs. Fitness browser suggest that CC_1156 mutants display cofitness with mutants deficient in CC_1168, CC_1166, CC_1161, CC_1160, CC_1159, CC_1158, and CC_1152 (S1 Table).

CC_1155 also contains the PFAM domain PF03739 (LptG/LptF) and codes for an integral membrane protein (TMHMM and Phobius predicts six transmembrane helices) (S2 Fig) which also might participate in the export of LPS or PSphLs.

CC_1154 encodes the *N*-acyltransferase bCerS that can convert 3-oxo-sphinganine or sphinganine to their respective *N*-acylated products [7].

CC_1153 is similar to the InterPro domain IPR029004 of GTP:adenosylcobinamide-phosphate guanilyltransferase (CobY) and to the biosynthesis protein for molybdopterine-guanine A (MobA) IPR025877. KEGG suggests that homologs are annotated as 2-phospho-L-lactate guanilyltransferase. This family is represented by CofC [10], a nucleotidyltransferase that participates in the biosynthesis of coenzyme F420.

CC_1152 has PFAM domains PF00483, PF1128, and PF2804 of nucleotidyltransferases. Many enzymes that transfer nucleotides to phosphosugars are included in this family. Fitness browser suggests that CC_1152-deficient mutant shows cofitness with mutants deficient in CC_1168, CC_1166, CC_1161, CC_1159, CC_1158, CC_1385 (predicted fructose-1,6-bisphosphatase), and CC_0001 (putative pyruvate, water dikinase/phosphoenolpyruvate synthetase) (S1 Table). Therefore, CC_1385 and CC_0001 seem to encode two critical gluconeogenesis enzymes.

In summary, homologs that encode transport proteins (CC_1168, CC_1166, CC_1156, CC_1155) might contribute to forming a complex transport system that moves PSphLs from their site of synthesis in the IM to their final destination in the outer layer of the OM in an analogous way to that in which LPS is transported. However, we did not investigate PSphL transport in more detail in this work. Instead, we focused on studying genes potentially coding for biosynthetic enzymes (CC_1161, CC_1160, CC_1159, CC_1158, CC_1153, CC_1152) that participate in PSphL biosynthesis.

**References**

**1.** Okuda S, Sherman DJ, Silhavy TJ, Ruiz N, Kahne D. Lipopolysaccharide transport and assembly at the outer membrane: the PEZ model. Nature Rev Microbiol. 2016; 14: 337–345.

**2.** Krogh A, Larsson B, von Heijne G, Sonnhammer EL. Predicting transmembrane protein topology with a hidden Markov model: application to complete genomes. J Mol Biol. 2001; 305(3): 567–580. doi: 10.1006/jmbi.2000.4315. PMID: 11152613

**3.** Käll L, Krogh A, Sonnhammer EL. Advantages of combined transmembrane topology and signal peptide prediction--the Phobius web server. Nucleic Acids Res. 2007; 35(Web Server issue): W429–W432. doi: [10.1093/nar/gkm256](https://doi.org/10.1093/nar/gkm256).

**4.** Okino N, Li M, Qu Q, Nakagawa T, Hayashi Y, Matsumoto M, et al. Two bacterial glycosphingolipid synthases responsible for the synthesis of glucuronosylceramide and α-galactosylceramide. J Biol Chem. 2020; 295: 10709–10725.

**5.** Mi W,Yoon LS, Ernst RK, Walz T, Liao M. Structural basis of MsbA-mediated lipopolysaccharide transport. Nature. 2017; 549: 233-237. doi: 10.1038/nature23649.

**6.** Padilla-Gómez J, Olea-Ozuna, RJ, Contreras-Martínez S, Morales-Tarré O, García-Soriano DA Sahonero-Canavesi DX, et al. Specialized acyl carrier protein used by serine palmitoyltransferase to synthesize sphingolipids in *Rhodobacteria*. Front Microbiol. 2022; 13: 961041. doi: 10.3389/fmicb.2022.961041.

**7.** Stankeviciute G, Tang P, Ashley B, Chamberlain JD, Hansen MEB, Coleman A., et al. Convergent evolution of bacterial ceramide synthesis. Nature Chem Biol. 2022; 18: 305-312. doi: 10.1038/s41589-021-00948-7.

**8.** Zik JJ, Yoon SH, Guan Z, Stankeviciute Skidmore G, Gudoor RR, Davies KM, et al. *Caulobacter* lipid A is conditionally dispensable in the absence of *fur* and in the presence of anionic sphingolipids. Cell Rep. 2022; 39:110888. doi: 10.1016/j.celrep.2022.110888.

**9.** Dhakephalkar T, Stukey GJ, Guan Z, Carman GM, Klein ER. Characterization of an evolutionary distinct bacterial ceramide kinase from *Caulobacter crescentus*. J Biol Chem. 2023; 299: 104894. doi: 10.1016/j.jbc.2023.104894.

**10.** Bashiri G, Antoney J, Jirgis ENM, Shah M, Ney B, Copp J, et al. A revised biosynthetic pathway for the cofactor F420 in prokaryotes. Nat Commun. 2019; 10: 1558. doi: 10.1038/s41467-019-09534-x.
