# Supplementary material for: Genes required for phosphosphingolipid formation in Caulobacter crescentus contribute to bacterial virulence
Source: PLoS Pathog. 2024 Aug 2;20(8):e1012401. doi: 10.1371/journal.ppat.1012401 (PMC11324152; doi:10.1371/journal.ppat.1012401)
Supplement: S1 Table — Cofitness data were taken from Price et al. [18]. Mutants in CC_1157 do not show high cofitness values with other mutants of the cluster. For CC_1155 and CC_1153 no data have been reported. CC_1165, CC_1164, CC_1163, CC_1162, or CC_1154 are indicated as aasR, cerR, acpR, spt, or cerS, respectively. (DOCX) [file ppat.1012401.s002.docx]

**S1 Table. Cofitness of mutants affected in genes putatively involved in PSphL biosynthesis and transport in *C. crescentus*.** Cofitness data were taken from Price *et al*. [18]. Mutants in CC_1157 do not show high cofitness values with other mutants of the cluster. For CC_1155 and CC_1153 no data have been reported. CC_1165, CC_1164, CC_1163, CC_1162, or CC_1154 are indicated as *aasR,* *cerR*, *acpR*, *spt*, or *cerS*, respectively.

| **Δ*CC_*** | ***1168*** | ***1167*** | ***1166*** | ***aasR*** | ***cerR*** | ***acpR*** | ***spt*** | ***1161*** | ***1160*** | ***1159*** | ***1158*** | ***1157*** | ***1156*** | ***1155*** | ***cerS*** | ***1153*** | ***1152*** | ***1385*** |
| --- | --- | --- | --- | --- | --- | --- | --- | --- | --- | --- | --- | --- | --- | --- | --- | --- | --- | --- |
| ***1168*** |  | 0.47 |  | 0.59 | 0.62 |  | 0.52 | 0.66 | 0.64 | 0.78 | 0.60 |  | 0.67 |  | 0.62 |  | 0.63 |  |
| ***1167*** | 0.47 |  |  | 0.64 | 0.51 | 0.57 | 0.57 |  | 0.49 | 0.54 | 0.66 |  |  |  | 0.64 |  |  |  |
| ***1166*** |  |  |  |  |  |  |  |  |  |  |  |  | 0.62 |  |  |  | 0.61 | 0.51 |
| ***aasR*** | 0.59 | 0.64 |  |  | 0.86 | 0.82 | 0.92 |  | 0.79 | 0.71 | 0.68 |  | 0.39 |  | 0.94 |  |  |  |
| ***1164*** | 0.62 | 0.51 |  | 0.86 |  | 0.69 | 0.80 |  | 0.86 | 0.72 | 0.65 |  | 0.49 |  | 0.86 |  |  |  |
| ***acpR*** |  | 0.57 |  | 0.82 | 0.69 |  | 0.88 |  | 0.60 | 0.52 | 0.60 |  |  |  | 0.73 |  |  |  |
| ***spt*** | 0.52 | 0.57 |  | 0.92 | 0.80 | 0.88 |  |  | 0.67 | 0.60 | 0.60 |  |  |  | 0.83 |  |  |  |
| ***1161*** | 0.66 |  |  |  |  |  |  |  | 0.44 | 0.79 | 0.48 |  | 0.61 |  | 0.42 |  | 0.82 | 0.67 |
| ***1160*** | 0.64 | 0.49 |  | 0.79 | 0.86 | 0.60 | 0.67 | 0.44 |  | 0.75 | 0.62 |  | 0.49 |  | 0.80 |  |  | 0.53 |
| ***1159*** | 0.78 | 0.54 |  | 0.71 | 0.72 | 0.52 | 0.60 | 0.79 | 0.75 |  | 0.72 |  | 0.60 |  | 0.75 |  | 0.74 |  |
| ***1158*** | 0.60 | 0.66 |  | 0.68 | 0.65 | 0.60 | 0.60 | 0.48 | 0.62 | 0.72 |  |  | 0.48 |  | 0.73 |  | 0.46 |  |
| ***1157*** |  |  |  |  |  |  |  |  |  |  |  |  |  |  |  |  |  |  |
| ***1156*** | 0.67 |  | 0.62 | 0.39 | 0.49 |  |  | 0.61 | 0.49 | 0.60 | 0.48 |  |  |  | 0.43 |  | 0.63 |  |
| ***1155*** |  |  |  |  |  |  |  |  |  |  |  |  |  |  |  |  |  |  |
| ***1154*** | 0.62 | 0.64 |  | 0.94 | 0.86 | 0.73 | 0.83 | 0.42 | 0.80 | 0.75 | 0.73 |  | 0.43 |  |  |  |  |  |
| ***1153*** |  |  |  |  |  |  |  |  |  |  |  |  |  |  |  |  |  |  |
| ***1152*** | 0.63 |  | 0.61 |  |  |  |  | 0.82 |  | 0.74 | 0.46 |  | 0.63 |  |  |  |  | 0.71 |
| ***1385*** |  |  | 0.51 |  |  |  |  | 0.67 |  | 0.53 |  |  |  |  |  |  | 0.71 |  |
