# Supplementary material for: Genes required for phosphosphingolipid formation in Caulobacter crescentus contribute to bacterial virulence
Source: PLoS Pathog. 2024 Aug 2;20(8):e1012401. doi: 10.1371/journal.ppat.1012401 (PMC11324152; doi:10.1371/journal.ppat.1012401)
Supplement: S2 Table — (DOCX) [file ppat.1012401.s003.docx]

**S2 Table.** **Orthologs of sphingolipids biosynthesis and transport proteins from *C. crescentus* in selected *Rhodobacteria* (α-, β-, and γ-proteobacteria).**

**S2A Table.** **Orthologs of sphingolipids biosynthesis and transport proteins from *C. crescentus* in *H. neptunium*.**

| **Proteins in**  ***C. crescentus*** | **Orthologs in**  ***H. neptunium***  **ATCC 15444** | **Coverage**  **(%)** | **E value** | **Identity**  **(%)** | **Similarity**  **(%)** |
| --- | --- | --- | --- | --- | --- |
| CC_1168 (CCNA_01226) | HNE_0203 | 91 | 2e-24 | 34 | 50 |
| CC_1167 (CCNA_01225) | HNE_1578 | 96 | 5e-79 | 39 | 54 |
| CC_1166 (CCNA_01224) | HNE_1594 | 93 | 1e-48 | 30 | 50 |
| CC_1165 (CCNA_01223) | HNE_1593 | 99 | 9e-160 | 44 | 62 |
| CC_1164 (CCNA_01222) | HNE_1592 | 88 | 1e-40 | 36 | 46 |
| CC_1163 (CCNA_01221) | HNE_1591 | 59 | 1e-12 | 39 | 68 |
| CC_1162 (CCNA_01220) | HNE_1590 | 95 | 2e-152 | 54 | 69 |
| CC_1161 (CCNA_01219) | HNE_1589 | 86 | 3e-37 | 30 | 51 |
| CC_1160 (CCNA_01218) | HNE_1588 | 90 | 4e-13 | 28 | 40 |
| CC_1159 (CCNA_01217) | HNE_1581 | 96 | 7e-97 | 41 | 56 |
| CC_1158 (CCNA_01216) | - | - | - | - | - |
| CC_1157 (CCNA_01215) | HNE_1582 | 96 | 2e-36 | 44 | 53 |
| CC_1156 (CCNA_01214) | HNE_1586 | 87 | 9e-44 | 33 | 51 |
| CC_1155 (CCNA_01213) | HNE_1587 | 94 | 3e-29 | 28 | 42 |
| CC_1154 (CCNA_01212) | HNE_1584 | 98 | 3e-90 | 41 | 55 |
| CC_1153 (CCNA_01211) | HNE_1583 | 83 | 6e-39 | 35 | 53 |
| CC_1152 (CCNA_01210) | HNE_1579 | 91 | 2e-71 | 47 | 64 |

Comparative sequence alignments of protein sequences from *C. crescentus* involved in the biosynthesis and transport of PSphLs with those from *H. neptunium*. The ORF names/accession numbers are as follows: *C. crescentus* CB15N (NA1000) (CCNA_01226/ACL94691.1; CCNA_01225/ACL94690.1; CCNA_01224/ACL94689.1; CCNA_01223/ACL94688.1; CCNA_01222/ACL94687.1; CCNA_01221/ ACL94686.1; CCNA_01220/ACL94685.1; CCNA_01219/ACL94684.1; CCNA_01218/ACL94683.3; CCNA_01217/ACL94682.1; CCNA_01216/ACL94681.1; CCNA_01215/ACL94680.1; CCNA_01214/ACL94679.1; CCNA_01213/ACL94678.1; CCNA_01212/ACL94677.1; CCNA_01211/ACL94676.3; CCNA_01210/ACL94675.1) and *H. neptunium* ATCC 15444 (HNE_0203/ABI78183.1; HNE_1578/ABI75740.1; HNE_1594/ABI77162.1; HNE_1593/ABI76156.1; HNE_1592/ABI75976.1; HNE_1591/ABI76753.1; HNE_1590/ABI75992.1; HNE_1589/ABI78724.1; HNE_1588/ABI77896.1; HNE_1581/ABI78751.1; HNE_1582/ABI76211.1; HNE_1586/ABI75469.1; HNE_1587/ABI76431.1; HNE_1584/ABI77389.1; HNE_1583/ABI75449.1; HNE_1579/ABI76316.1).

**S2B Table.** **Orthologs of sphingolipids biosynthesis and transport proteins from *C. crescentus* in *S. wittichii*.**

| **Proteins in**  ***C. crescentus*** | **Orthologs in**  ***S. wittichii* RW1** | **Coverage**  **(%)** | **E value** | **Identity**  **(%)** | **Similarity**  **(%)** |
| --- | --- | --- | --- | --- | --- |
| CC_1168 (CCNA_01226) | Swit_2904 | 73 | 2e-06 | 23 | 40 |
| CC_1167 (CCNA_01225) | Swit_4739  Swit_4514 | 98  96 | 8e-99  6e-97 | 43  44 | 57  58 |
| CC_1166 (CCNA_01224) | Swit_2493 | 83 | 8e-30 | 30 | 47 |
| CC_1165 (CCNA_01223) | Swit_2559 | 98 | 3e-168 | 46 | 63 |
| CC_1164 (CCNA_01222) | Swit_3897 | 95 | 5e-50 | 40 | 47 |
| CC_1163 (CCNA_01221) | Swit_3899 | 68 | 3e-10 | 32 | 54 |
| CC_1162 (CCNA_01220) | Swit_3900 | 97 | 5e-157 | 53 | 73 |
| CC_1161 (CCNA_01219) | Swit_3902 | 96 | 8e-44 | 39 | 50 |
| CC_1160 (CCNA_01218) | Swit_3903 | 91 | 5e-18 | 25 | 45 |
| CC_1159 (CCNA_01217) | Swit_1307 | 95 | 7e-102 | 45 | 59 |
| CC_1158 (CCNA_01216) | - | - | - | - | - |
| CC_1157 (CCNA_01215) | Swit_1308 | 93 | 2e-50 | 54 | 64 |
| CC_1156 (CCNA_01214) | Swit_3905 | 96 | 8e-42 | 30 | 46 |
| CC_1155 (CCNA_01213) | Swit_3906 | 95 | 3e-25 | 28 | 42 |
| CC_1154 (CCNA_01212) | Swit_3908 | 97 | 5e-109 | 45 | 58 |
| CC_1153 (CCNA_01211) | Swit_1306 | 52 | 2e-29 | 40 | 55 |
| CC_1152 (CCNA_01210) | Swit_1309 | 96 | 4e-83 | 54 | 67 |

Comparative sequence alignments of protein sequences from *C. crescentus* involved in the biosynthesis and transport of PSphLs with those from *S. wittichii*. The ORF names/accession numbers are as follows: *C. crescentus* CB15N (NA1000) (CCNA_01226/ACL94691.1; CCNA_01225/ACL94690.1; CCNA_01224/ACL94689.1; CCNA_01223/ACL94688.1; CCNA_01222/ACL94687.1; CCNA_01221/ ACL94686.1; CCNA_01220/ACL94685.1; CCNA_01219/ACL94684.1; CCNA_01218/ACL94683.3; CCNA_01217/ACL94682.1; CCNA_01216/ACL94681.1; CCNA_01215/ACL94680.1; CCNA_01214/ACL94679.1; CCNA_01213/ACL94678.1; CCNA_01212/ACL94677.1; CCNA_01211/ACL94676.3; CCNA_01210/ACL94675.1) and *S. wittichii* RW1 (Swit_2904/ABQ69256.1; Swit_4739/ABQ71076.1; Swit_4514/ABQ70852.1; Swit_2493/ABQ68852.1; Swit_2559/ABQ68917.1; Swit_3897/ABQ70242.1; Swit_3899/ABQ70244.1; Swit_3900/ABQ70245.1; Swit_3902/ABQ70247.1; Swit_3903/ABQ70248.1; Swit_1307/ABQ67672.1; Swit_1308/ABQ67673.1; Swit_3905/ABQ70250.1; Swit_3906/ABQ70251.1; Swit_3908/ABQ70253.1; Swit_1306/ABQ67671.1; Swit_1309/ABQ67674.1).

**S2C Table.** **Orthologs of sphingolipids biosynthesis and transport proteins from *C. crescentus* in *Z. mobilis*.**

| **Proteins in**  ***C. crescentus*** | **Orthologs in**  ***Z. mobilis* ZM4** | **Coverage**  **(%)** | **E value** | **Identity**  **(%)** | **Similarity**  **(%)** |
| --- | --- | --- | --- | --- | --- |
| CC_1168 (CCNA_01226) | ZMO0277 | 83 | 1e-08 | 21 | 39 |
| CC_1167 (CCNA_01225) | ZMO1734  ZMO1777 | 99  98 | 4e-83  4e-79 | 38  34 | 55  54 |
| CC_1166 (CCNA_01224) | ZMO1443 | 98 | 2e-69 | 30 | 50 |
| CC_1165 (CCNA_01223) | ZMO0704 | 98 | 1e-168 | 45 | 63 |
| CC_1164 (CCNA_01222) | ZMO0205 | 96 | 4e-43 | 31 | 48 |
| CC_1163 (CCNA_01221) | ZMO2012 | 66 | 2e-12 | 38 | 57 |
| CC_1162 (CCNA_01220) | ZMO1270 | 97 | 1e-155 | 52 | 72 |
| CC_1161 (CCNA_01219) | ZMO1390 | 95 | 1e-51 | 36 | 52 |
| CC_1160 (CCNA_01218) | ZMO1391 | 86 | 2e-22 | 29 | 51 |
| CC_1159 (CCNA_01217) | - | - | - | - | - |
| CC_1158 (CCNA_01216) | - | - | - | - | - |
| CC_1157 (CCNA_01215) | - | - | - | - | - |
| CC_1156 (CCNA_01214) | ZMO1393 | 90 | 4e-37 | 25 | 45 |
| CC_1155 (CCNA_01213) | ZMO1394 | 93 | 2e-26 | 26 | 42 |
| CC_1154 (CCNA_01212) | ZMO1400 | 98 | 2e-97 | 40 | 57 |
| CC_1153 (CCNA_01211) | ZMO0107 | 84 | 3e-30 | 27 | 46 |
| CC_1152 (CCNA_01210) | ZMO1100 | 24 | 3e-15 | 46 | 70 |

Comparative sequence alignments of protein sequences from *C. crescentus* involved in the biosynthesis and transport of PSphLs with those from *Z. mobilis*. The ORF names/accession numbers are as follows: *C. crescentus* CB15N (NA1000) (CCNA_01226/ACL94691.1; CCNA_01225/ACL94690.1; CCNA_01224/ACL94689.1; CCNA_01223/ACL94688.1; CCNA_01222/ACL94687.1; CCNA_01221/ ACL94686.1; CCNA_01220/ACL94685.1; CCNA_01219/ACL94684.1; CCNA_01218/ACL94683.3; CCNA_01217/ACL94682.1; CCNA_01216/ACL94681.1; CCNA_01215/ACL94680.1; CCNA_01214/ACL94679.1; CCNA_01213/ACL94678.1; CCNA_01212/ACL94677.1; CCNA_01211/ACL94676.3; CCNA_01210/ACL94675.1) and *Z. mobilis* ZM4 (ZMO0277/AAV88901.1; ZMO1734/AAV90358.1; ZMO1777/ AAV90401.2; ZMO1443/AAV90067.2; ZMO0704/AAV89328.2; ZMO0205/AAV88829.1; ZMO2012/ADK75091.1; ZMO1270/AAV89894.1; ZMO1390/AAV90014.2; ZMO1391/AAV90015.1; ZMO1393/AAV90017.2; ZMO1394/AAV90018.1; ZMO1400/AAV90024.1; ZMO0107/AAV88731.1; ZMO1100/AAV89724.1).

**S2D Table.** **Orthologs of sphingolipids biosynthesis and transport proteins from *C. crescentus* in *N. eutropha*.**

| **Proteins in**  ***C. crescentus*** | **Orthologs in**  ***N. eutropha* C91** | **Coverage**  **(%)** | **E value** | **Identity**  **(%)** | **Similarity**  **(%)** |
| --- | --- | --- | --- | --- | --- |
| CC_1168 (CCNA_01226) | - | - | - | - | - |
| CC_1167 (CCNA_01225) | - | - | - | - | - |
| CC_1166 (CCNA_01224) | - | - | - | - | - |
| CC_1165 (CCNA_01223) | Neut_0458 | 98 | 3e-156 | 43 | 60 |
| CC_1164 (CCNA_01222) | Neut_0459 | 85 | 4e-52 | 38 | 52 |
| CC_1163 (CCNA_01221) | Neut_0460 | 68 | 8e-18 | 44 | 71 |
| CC_1162 (CCNA_01220) | Neut_0461 | 97 | 5e-138 | 51 | 66 |
| CC_1161 (CCNA_01219) | Neut_1106 | 86 | 2e-38 | 32 | 48 |
| CC_1160 (CCNA_01218) | Neut_1105 | 89 | 2e-22 | 25 | 45 |
| CC_1159 (CCNA_01217) | Neut_1104 | 96 | 5e-75 | 41 | 55 |
| CC_1158 (CCNA_01216) | - | - | - | - | - |
| CC_1157 (CCNA_01215) | Neut_0267 | 96 | 1e-39 | 44 | 58 |
| CC_1156 (CCNA_01214) | Neut_1102 | 83 | 1e-09 | 24 | 42 |
| CC_1155 (CCNA_01213) | Neut_1103 | 96 | 6e-15 | 33 | 37 |
| CC_1154 (CCNA_01212) | Neut_1100 | 100 | 5e-105 | 41 | 58 |
| CC_1153 (CCNA_01211) | Neut_1101 | 85 | 7e-36 | 33 | 49 |
| CC_1152 (CCNA_01210) | Neut_0268 | 93 | 5e-62 | 44 | 62 |

Comparative sequence alignments of protein sequences from *C. crescentus* involved in the biosynthesis and transport of PSphLs with those from *N. eutropha*. The ORF names/accession numbers are as follows: *C. crescentus* CB15N (NA1000) (CCNA_01226/ACL94691.1; CCNA_01225/ACL94690.1; CCNA_01224/ACL94689.1; CCNA_01223/ACL94688.1; CCNA_01222/ACL94687.1; CCNA_01221/ ACL94686.1; CCNA_01220/ACL94685.1; CCNA_01219/ACL94684.1; CCNA_01218/ACL94683.3; CCNA_01217/ACL94682.1; CCNA_01216/ACL94681.1; CCNA_01215/ACL94680.1; CCNA_01214/ACL94679.1; CCNA_01213/ACL94678.1; CCNA_01212/ACL94677.1; CCNA_01211/ACL94676.3; CCNA_01210/ACL94675.1) and *N. eutropha* C91 (Neut_0458/ABI58735.1; Neut_0459/ABI58736.1; Neut_0460/ABI58737.1; Neut_0461/ABI58738.1; Neut_1106/ABI59361.1; Neut_1105/ABI59360.1; Neut_1104/ABI59359.1; Neut_0267/ABI58551.1; Neut_1102/ABI59357.1; Neut_1103/ABI59358.1; Neut_1100/ABI59355.1; Neut_1101/ABI59356.1; Neut_0268/ABI58552.1).

**S2E Table.** **Orthologs of sphingolipids biosynthesis and transport proteins from *C. crescentus* in *A. vinelandii*.**

| **Proteins in**  ***C. crescentus*** | **Orthologs in**  ***A. vinelandii* DJ** | | **Coverage**  **(%)** | **E value** | **Identity**  **(%)** | **Similarity**  **(%)** |
| --- | --- | --- | --- | --- | --- | --- |
| CC_1168 (CCNA_01226) | | - | - | - | - | - |
| CC_1167 (CCNA_01225) | | - | - | - | - | - |
| CC_1166 (CCNA_01224) | | - | - | - | - | - |
| CC_1165 (CCNA_01223) | | Avin_44270 (pseudogen) | - | - | - | - |
| CC_1164 (CCNA_01222) | | Avin_44260 | 86 | 1e-43 | 38 | 50 |
| CC_1163 (CCNA_01221) | | Avin_44250 | 58 | 2e-14 | 46 | 72 |
| CC_1162 (CCNA_01220) | | Avin_44240 | 89 | 2e-149 | 57 | 71 |
| CC_1161 (CCNA_01219) | | Avin_44370 | 91 | 1e-27 | 32 | 44 |
| CC_1160 (CCNA_01218) | | Avin_44360 | 90 | 1e-20 | 30 | 43 |
| CC_1159 (CCNA_01217) | | Avin_44350 | 96 | 1e-85 | 41 | 57 |
| CC_1158 (CCNA_01216) | | - | - | - | - | - |
| CC_1157 (CCNA_01215) | | - | - | - | - | - |
| CC_1156 (CCNA_01214) | | Avin_44330 | 74 | 1e-06 | 24 | 39 |
| CC_1155 (CCNA_01213) | | Avin_44340 | 83 | 9e-13 | 24 | 39 |
| CC_1154 (CCNA_01212) | | Avin_44290 | 95 | 7e-111 | 47 | 64 |
| CC_1153 (CCNA_01211) | | Avin_44300 | 83 | 8e-37 | 37 | 51 |
| CC_1152 (CCNA_01210) | | Avin_44310 | 94 | 1e-83 | 54 | 69 |

Comparative sequence alignments of protein sequences from *C. crescentus* involved in the biosynthesis and transport of PSphLs with those from *A. vinelandii*. The ORF names/accession numbers are as follows: *C. crescentus* CB15N (NA1000) (CCNA_01226/ACL94691.1; CCNA_01225/ACL94690.1; CCNA_01224/ACL94689.1; CCNA_01223/ACL94688.1; CCNA_01222/ACL94687.1; CCNA_01221/ ACL94686.1; CCNA_01220/ACL94685.1; CCNA_01219/ACL94684.1; CCNA_01218/ACL94683.3; CCNA_01217/ACL94682.1; CCNA_01216/ACL94681.1; CCNA_01215/ACL94680.1; CCNA_01214/ACL94679.1; CCNA_01213/ACL94678.1; CCNA_01212/ACL94677.1; CCNA_01211/ACL94676.3; CCNA_01210/ACL94675.1) and *A. vinelandii* DJ (Avin_44260/ACO80547.1; Avin_44250/ACO80546.1; Avin_44240/ACO80545.1; Avin_44370/ACO80555.1; Avin_44360/ACO80554.1; Avin_44350/ACO80553.1; Avin_44330/ACO80551.1; Avin_44340/ACO80552.1; Avin_44290/ACO80548.1; Avin_44300/ACO80549.1; Avin_44310/ACO80550.1).

**S2F Table.** **Orthologs of sphingolipids biosynthesis and transport proteins from *C. crescentus* in *P. luteola*.**

| **Proteins in**  ***C. crescentus*** | **Orthologs in**  ***P. luteola***  **FOB45_16280** | **Coverage**  **(%)** | **E value** | **Identity**  **(%)** | **Similarity**  **(%)** |
| --- | --- | --- | --- | --- | --- |
| CC_1168 (CCNA_01226) | - | - | - | - | - |
| CC_1167 (CCNA_01225) | - | - | - | - | - |
| CC_1166 (CCNA_01224) | - | - | - | - | - |
| CC_1165 (CCNA_01223) | FOB45_16295 | 99 | 2e-172 | 44 | 63 |
| CC_1164 (CCNA_01222) | FOB45_16290 | 88 | 1e-50 | 40 | 54 |
| CC_1163 (CCNA_01221) | FOB45_16285 | 47 | 3e-12 | 46 | 73 |
| CC_1162 (CCNA_01220) | FOB45_16280 | 90 | 4e-153 | 55 | 73 |
| CC_1161 (CCNA_01219) | FOB45_16340 | 82 | 9e-32 | 30 | 46 |
| CC_1160 (CCNA_01218) | FOB45_16335 | 91 | 1e-20 | 29 | 41 |
| CC_1159 (CCNA_01217) | FOB45_16330 | 96 | 1e-80 | 40 | 57 |
| CC_1158 (CCNA_01216) | - | - | - | - | - |
| CC_1157 (CCNA_01215) | FOB45_16300 | 96 | 1e-45 | 45 | 60 |
| CC_1156 (CCNA_01214) | FOB45_16320 | 51 | 8e-08 | 24 | 42 |
| CC_1155 (CCNA_01213) | FOB45_16325 | 81 | 8e-14 | 25 | 39 |
| CC_1154 (CCNA_01212) | FOB45_16305 | 94 | 8e-111 | 45 | 62 |
| CC_1153 (CCNA_01211) | FOB45_16310 | 85 | 9e-39 | 35 | 50 |
| CC_1152 (CCNA_01210) | FOB45_16315 | 94 | 6e-83 | 50 | 67 |

Comparative sequence alignments of protein sequences from *C. crescentus* involved in the biosynthesis and transport of PSphLs with those from *P. luteola*. The ORF names/accession numbers are as follows: *C. crescentus* CB15N (NA1000) (CCNA_01226/ACL94691.1; CCNA_01225/ACL94690.1; CCNA_01224/ACL94689.1; CCNA_01223/ACL94688.1; CCNA_01222/ACL94687.1; CCNA_01221/ ACL94686.1; CCNA_01220/ACL94685.1; CCNA_01219/ACL94684.1; CCNA_01218/ACL94683.3; CCNA_01217/ACL94682.1; CCNA_01216/ACL94681.1; CCNA_01215/ACL94680.1; CCNA_01214/ACL94679.1; CCNA_01213/ACL94678.1; CCNA_01212/ACL94677.1; CCNA_01211/ACL94676.3; CCNA_01210/ACL94675.1) and *P. luteola* FOB45_16280 (FOB45_16295/QEU29237.1; FOB45_16290/ QEU29236.1; FOB45_16285/QEU29235.1; FOB45_16280/QEU31312.1; FOB45_16340/QEU29244.1; FOB45_16335/QEU29243.1; FOB45_16330/QEU29242.1; FOB45_16300/QEU29238.1; FOB45_16320/ QEU29240.1; FOB45_16325/QEU29241.1; FOB45_16305/QEU31313.1; FOB45_16310/QEU29239.1; FOB45_16315/QEU31314.1).
