# Supplementary material for: Genes required for phosphosphingolipid formation in Caulobacter crescentus contribute to bacterial virulence
Source: PLoS Pathog. 2024 Aug 2;20(8):e1012401. doi: 10.1371/journal.ppat.1012401 (PMC11324152; doi:10.1371/journal.ppat.1012401)
Supplement: S3 Table — (DOCX) [file ppat.1012401.s004.docx]

**S3 Table**. **Construction of *C. crescentus* knock-out mutants in potential sphingolipid biosynthesis genes.**

____________________________________________________________________________________

*C. crescentus* Mutant characteristics and Restriction PCR

mutant oligonucleotide primers used for construction site product

(bp)

____________________________________________________________________________________

**SPG14 *cc_1152* in frame deletion**

primer pair for upstream region:

CAAAAAGCTTCCAAGGCCGCCGCCGACATCG HindIII

CAAAGGATCCAACCGGCTGCATGACGTTCTGG BamHI 614

primer pair for downstream region:

CAAAGGATCCCAGGAGGCCGAGGCGGTCTAGG BamHI

CAAAGAATTCCCTGGCTGACACCCCTGACTGG EcoRI 618

In mutant SPG14 (Δ*1152*) a deletion of 729 bp, coding for amino acid residues 5-247 of a predicted nucleotidyltransferase family protein (253 amino acid residues in total), is replaced by a hexanucleotide providing a BamHI restriction site and coding for G and S.

**SPG15 *cc_1153* in frame deletion**

primer pair for upstream region:

CAAAAAGCTTCCCCGCCGATGGTCTTGATGG HindIII

CAAGGATCCACCCGCGAGGATCAGGGCCTTG BamHI 739

primer pair for downstream region:

CAAAGGATCCCTGGTGGGCGAGGCGAAAAGC BamHI

CAAAGAATTCAGGTCGCGCGCCTGTCGCTGC EcoRI 748

In mutant SPG15 (Δ*1153*) a deletion of 705 bp, coding for amino acid residues 44-278 of a predicted MobA-like NTP transferase domain protein (291 amino acid residues in total), is replaced by a hexanucleotide providing a BamHI restriction site and coding for G and S.

**SPG18 *cc_1161* deletion**

primer pair for upstream region:

CAAAAAGCTTCCTTGAAGGCGGCGCTGGACG HindIII

CAAAGGATCCTGGGAGAGCTCGGCCGCGACG BamHI 843

primer pair for downstream region:

CAAAGGATCCCTGGAGAGAGCGGCGGCAAGC BamHI

CAAAGAATTCGAAGACTTGGCCAAGCGCCTGC EcoRI 834

In mutant SPG18 (Δ*1161*) a deletion of 813 bp, coding for amino acid residues 14-284 of a predicted cytosolic protein (882 amino acid residues in total), is replaced by a hexanucleotide providing a BamHI restriction site and coding for G and S.
