# Supplementary material for: Genes required for phosphosphingolipid formation in Caulobacter crescentus contribute to bacterial virulence
Source: PLoS Pathog. 2024 Aug 2;20(8):e1012401. doi: 10.1371/journal.ppat.1012401 (PMC11324152; doi:10.1371/journal.ppat.1012401)
Supplement: S4 Table — Sites for recognition by restriction enzymes are underlined. (DOCX) [file ppat.1012401.s005.docx]

**S4 Table.** **Oligonucleotides used for amplification of different sphingolipid biosynthesis genes. Sites for recognition by restriction enzymes are underlined.**

| **Primer Sequences** | **(5´-3´)** |
| --- | --- |
|  |  |
| **Primers for expression plasmids** |  |
| oLOP444 | AGGAATACATATGCAGCCGGTTAAGACCCTTATTCTC |
| oLOP445 | ACTGGGATCCCTAGACCGCCTCGGCCTCCTGG |
| oLOP446 | AGGAATACATATGGGTTCTGGAGGCCAACAAGGC |
| oLOP447 | ACTGGAATTCTTAGCCTTTGAAATGTAAAGGGCTTTTCGC |
| oLOP448 | AGGAATACATATGTCCATTTATCGCATCGCCCACC |
| oLOP449 | ACTGGGATCCTCAGGCGGCCTCGGGG |
| oLOP450 | AGGAATACATATGAGTAGTGAAGTTCAAAAAGGGCCG |
| oLOP451 | ACTGGGATCCTCATTTCGCCAGCCAGGACTG |
| oLOP452 | AGGAATACATATGCTTCGTCGTGCACGCCATC |
| oLOP453 | ACTGGGTACCTCATCCGACCAGGAACCGCAAG |
| oLOP454 | AGGAATACATATGAGCCGCCTGCGCGG |
| oLOP455 | ACTGGGTACCCTATGCGGCTTGCCGCCGC |
