# Supplementary material for: Genes required for phosphosphingolipid formation in Caulobacter crescentus contribute to bacterial virulence
Source: PLoS Pathog. 2024 Aug 2;20(8):e1012401. doi: 10.1371/journal.ppat.1012401 (PMC11324152; doi:10.1371/journal.ppat.1012401)
Supplement: S5 Table — For details see Materials and Methods in main text. (DOCX) [file ppat.1012401.s006.docx]

**S5 Table.** **Construction of different expression plasmids.**

For details see Experimental Procedures in main text.

| Plasmid | ORFs amplified | Oligonucleotides used | Restricted with | Cloned into plasmid restricted with ( ) |
| --- | --- | --- | --- | --- |
| pRJ14 | *cc_1152* | oLOP444/oLOP445 | NdeI/BamHI | pET17b (NdeI/BamHI) |
| pRJ15 | *cc_1153* | oLOP446/oLOP447 | NdeI/EcoRI | pET17b (NdeI/EcoRI) |
| pRJ16 | *cc_1158* | oLOP448/oLOP449 | NdeI/BamHI | pET17b (NdeI/BamHI) |
| pRJ17 | *cc_1159* | oLOP450/oLOP451 | NdeI/BamHI | pET17b (NdeI/BamHI) |
| pRJ18 | *cc_1160* | oLOP452/oLOP453 | NdeI/KpnI | pET17b (NdeI/KpnI) |
| pRJ19 | *cc_1159/cc_1160* | oLOP450/oLOP453 | NdeI/KpnI | pET17b (NdeI/KpnI) |
| pRJ20 | *cc_1161* | oLOP454/oLOP455 | NdeI/KpnI | pET17b (NdeI/KpnI) |
| pRJ21 | *cc_1152* | Recloned from pRJ14 | NdeI/EcoRI | pBXMCS-2 (NdeI/EcoRI) |
| pRJ22 | *cc_1153* | Recloned from pRJ15 | NdeI/EcoRI | pBXMCS-2 (NdeI/EcoRI) |
| pRJ23 | *cc_1158* | Recloned from pRJ16 | NdeI/EcoRI | pBXMCS-2 (NdeI/EcoRI) |
| pRJ24 | *cc_1159* | Recloned from pRJ17 | NdeI/EcoRI | pBXMCS-2 (NdeI/EcoRI) |
| pRJ25 | *cc_1160* | Recloned from pRJ18 | NdeI/EcoRI | pBXMCS-2 (NdeI/EcoRI) |
| pRJ26 | *cc_1159/cc_1160* | Recloned from pRJ19 | NdeI/EcoRI | pBXMCS-2 (NdeI/EcoRI) |
| pRJ27 | *cc_1161* | Recloned from pRJ20 | NdeI/EcoRI | pBXMCS-2 (NdeI/EcoRI) |
