# Supplementary figures and images for: Genes required for phosphosphingolipid formation in Caulobacter crescentus contribute to bacterial virulence
Source: PLoS Pathog. 2024 Aug 2;20(8):e1012401. doi: 10.1371/journal.ppat.1012401 (PMC11324152; doi:10.1371/journal.ppat.1012401)

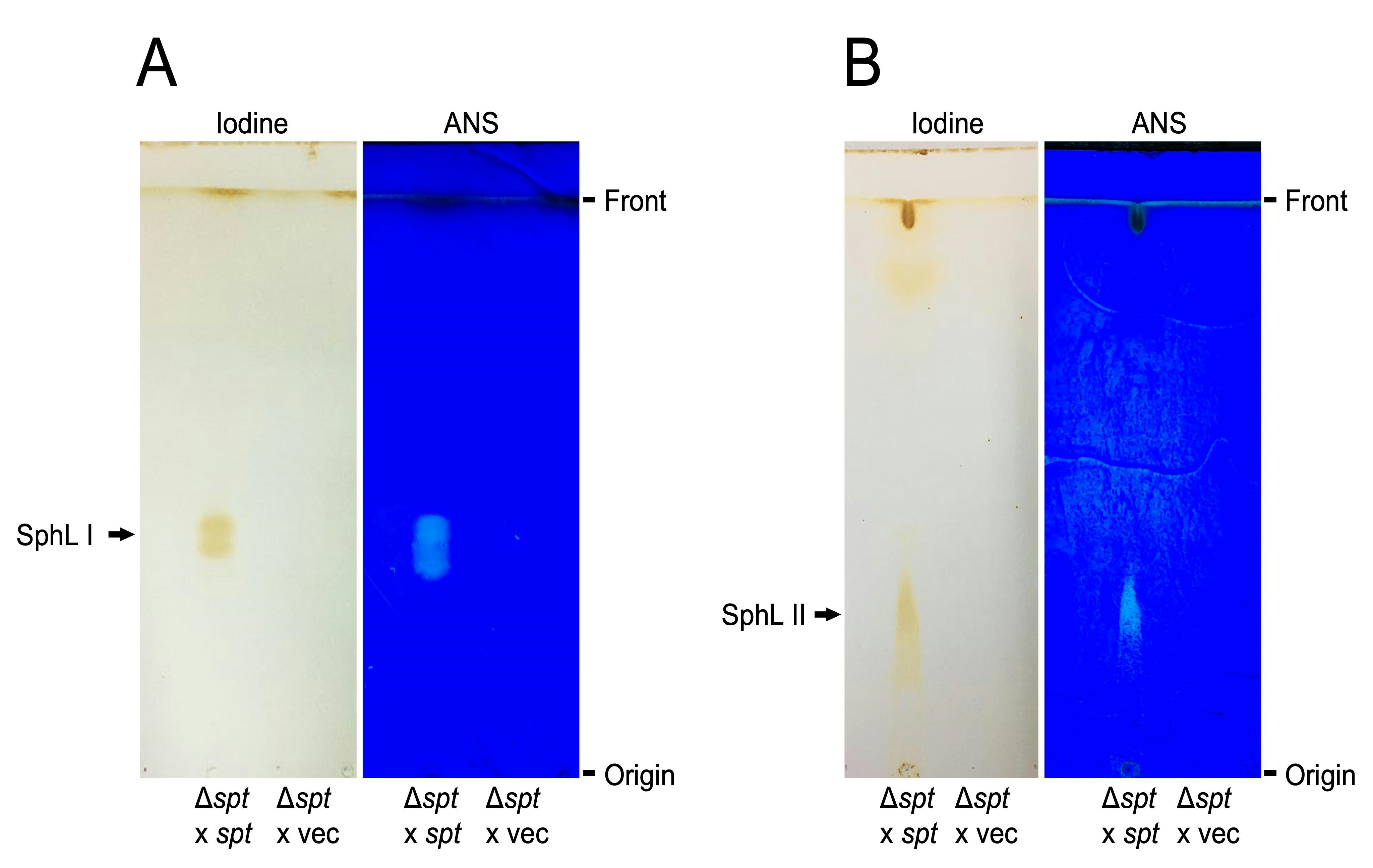

Supplement: S1 Fig — Lipid extracts from large cultures (2 l) of the spt-deficient mutant harboring the spt gene in trans (Δspt x spt) or of the spt-deficient mutant harboring the empty vector pRXMCS-2 (Δspt x vec) of C. crescentus were obtained, separated by preparative TLC, and visualized on exposure to iodine vapor. From areas that contained SphL I or II, silica gel was scraped, lipids were extracted, and aliquots were reanalyzed by TLC in order to assess their purity. As visualized after iodine or ANS staining, fractions enriched in SphL I (panel A) or SphL II (panel B) were obtained from the SphL-producing Δspt x spt strain while no analogous lipids were observed following the same extraction procedures carried out on the Δspt x vec strain of C. crescentus. (TIF) [file ppat.1012401.s007.tif]

cc\_1168

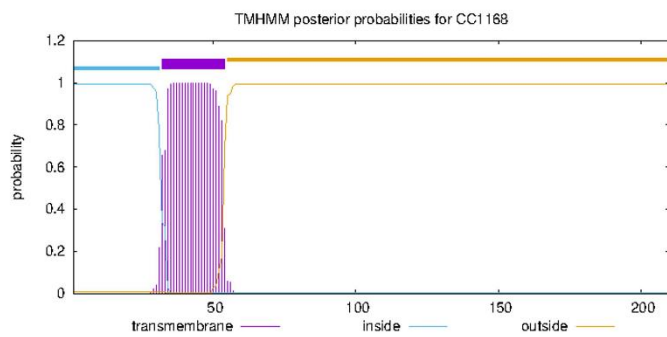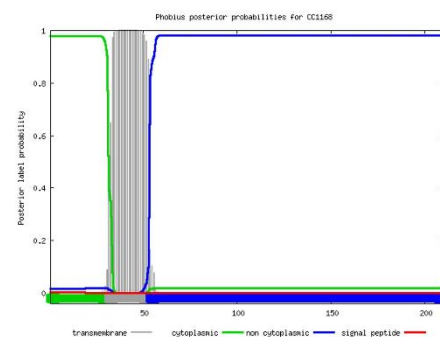

cc\_1166

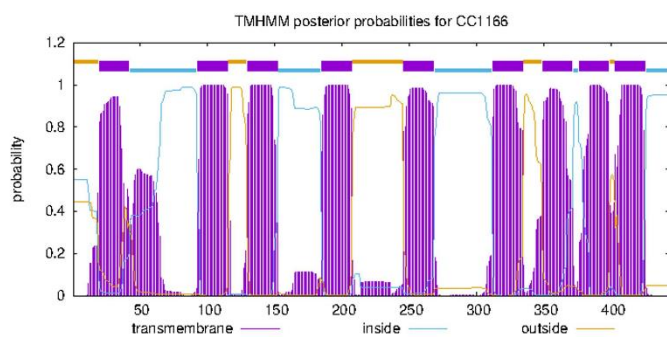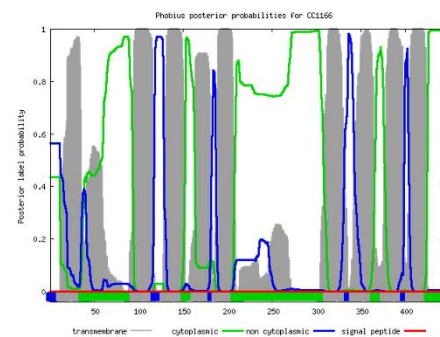

cc\_1160

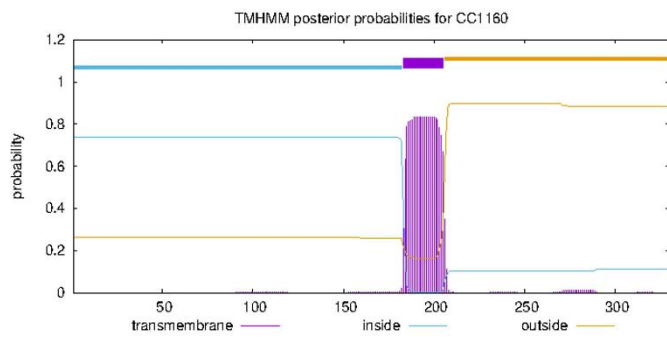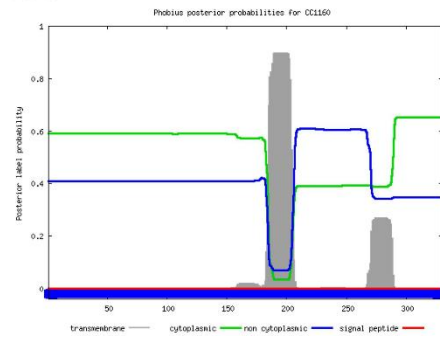

## CC\_1159

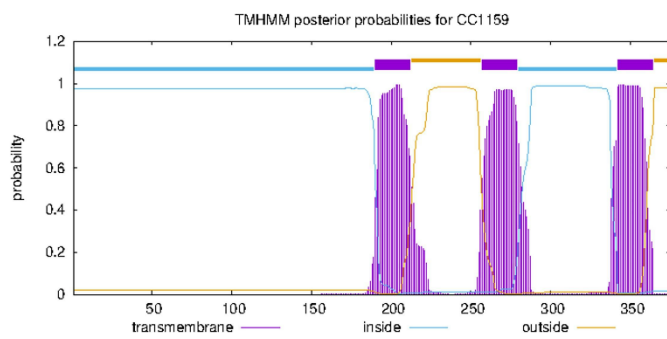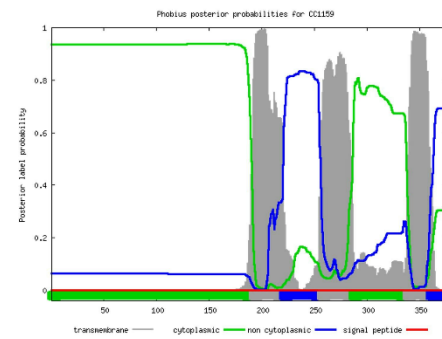

## CC\_1156

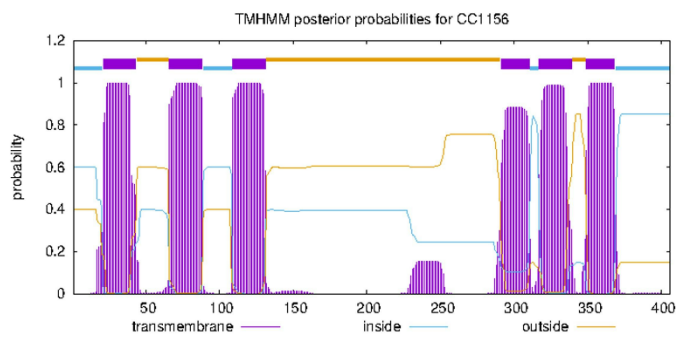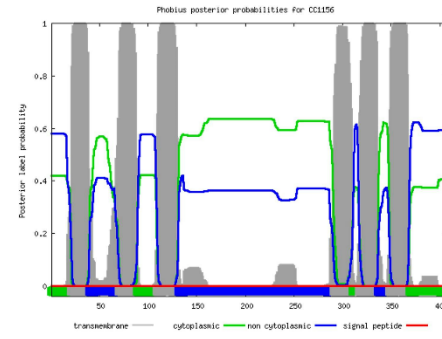

## CC\_1155

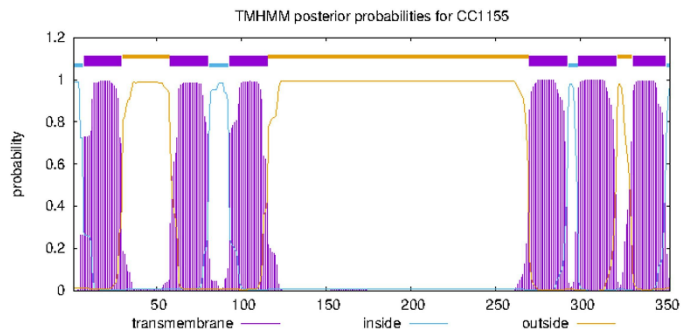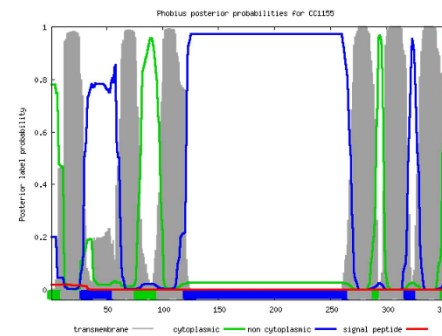

Supplement: S2 Fig — (PDF) [file ppat.1012401.s008.pdf]

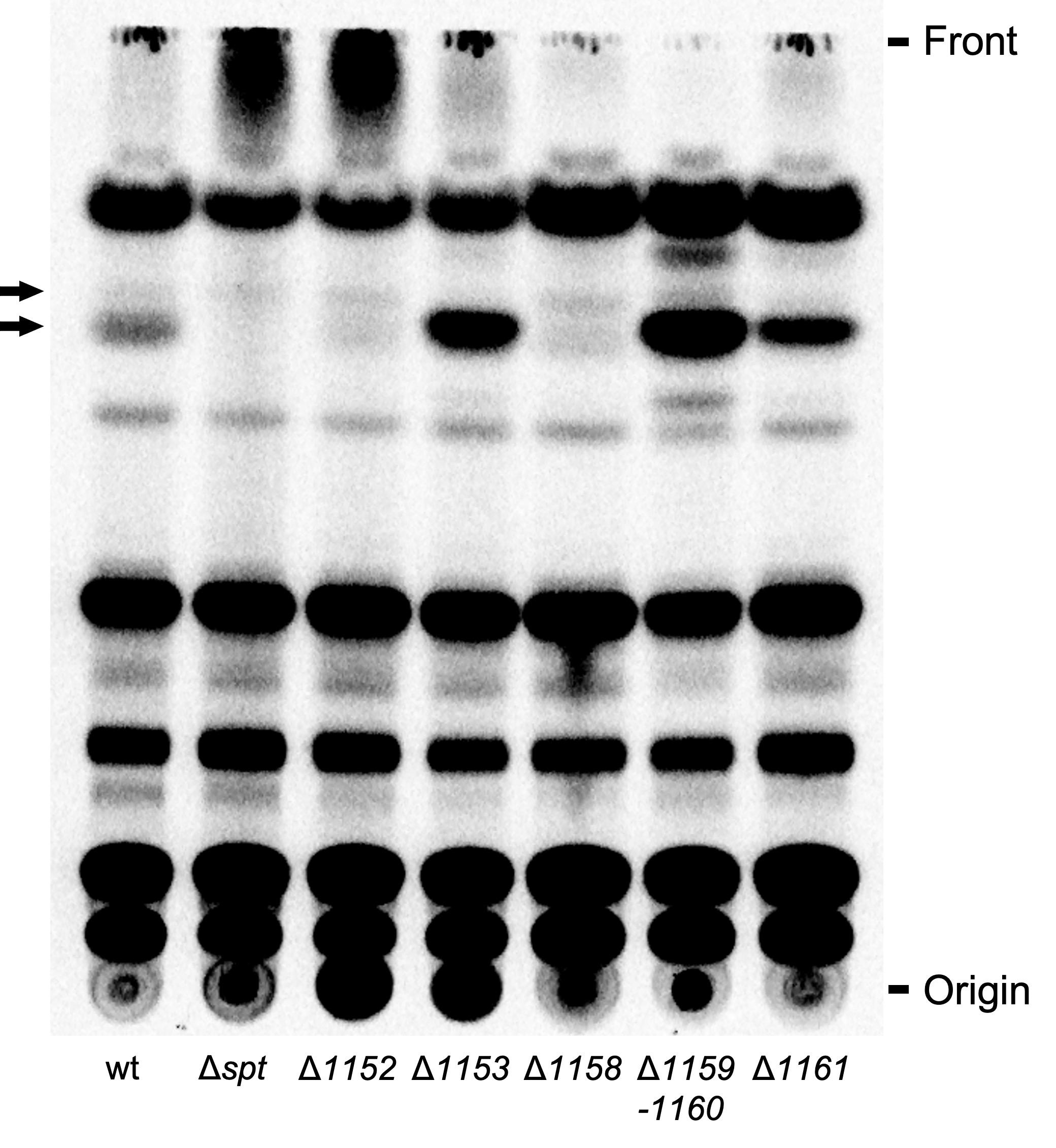

Supplement: S3 Fig — Different C. crescentus strains [wild-type strain (wt), spt-deficient mutant (Δspt), mutant SPG14 deficient in CC_1152 (Δ1152), mutant SPG15 deficient in CC_1153 (Δ1153), mutant SPG11 deficient in CC_1158 (Δ1158), mutant SPG09 deficient in CC_1159–1160 (Δ1159–1160), and mutant SPG18 deficient in CC_1161 (Δ1161)] were cultured in complex medium in the presence of 14C-acetate. After harvesting cells, lipids were extracted, separated by TLC in chloroform/methanol/ammonium hydroxide (40:10:1) and developed chromatograms were analyzed by phosphorimaging. Arrows indicate dihydroceramides formed by C. crescentus. (TIF) [file ppat.1012401.s009.tif]

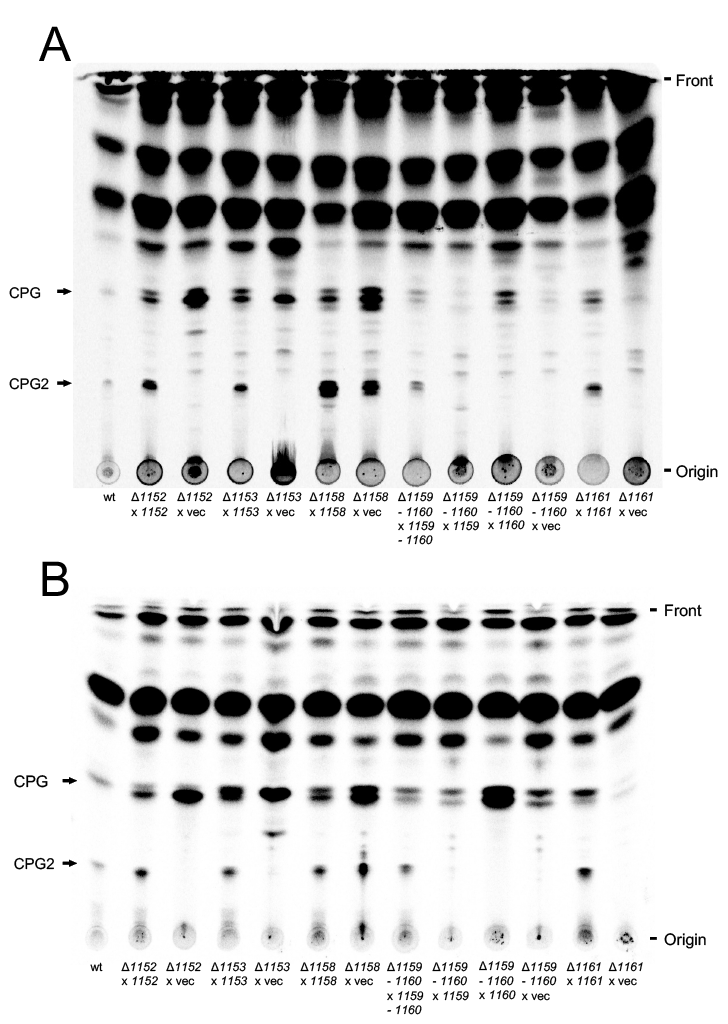

Supplement: S4 Fig — Mutants of C. crescentus deficient in CC_1152, CC_1153, CC_1158, CC_1159–1160, or CC_1161 carrying the respective intact gene in the xylose-inducible plasmid pBXMCS-2 (Δ1152 x 1152, Δ1153 x 1153, Δ1158 x 1158, Δ1159–1160 x 1159, Δ1159–1160 x 1160, Δ1159–1160 x 1159–1160, Δ1161 x 1161) or carrying the empty pBXMCS-2 vector (vec) in trans were radiolabeled with 14C-acetate (A) or 33P-phosphate (B) for 16 h. At the end of the labeling period, cells were harvested, lipids were extracted, separated by TLC in chloroform/methanol/acetic acid/water (8:3:2:1) and developed chromatograms were subjected to autoradiography. Arrows indicate PSphLs (CPG and CPG2) formed by C. crescentus. (TIF) [file ppat.1012401.s010.tif]

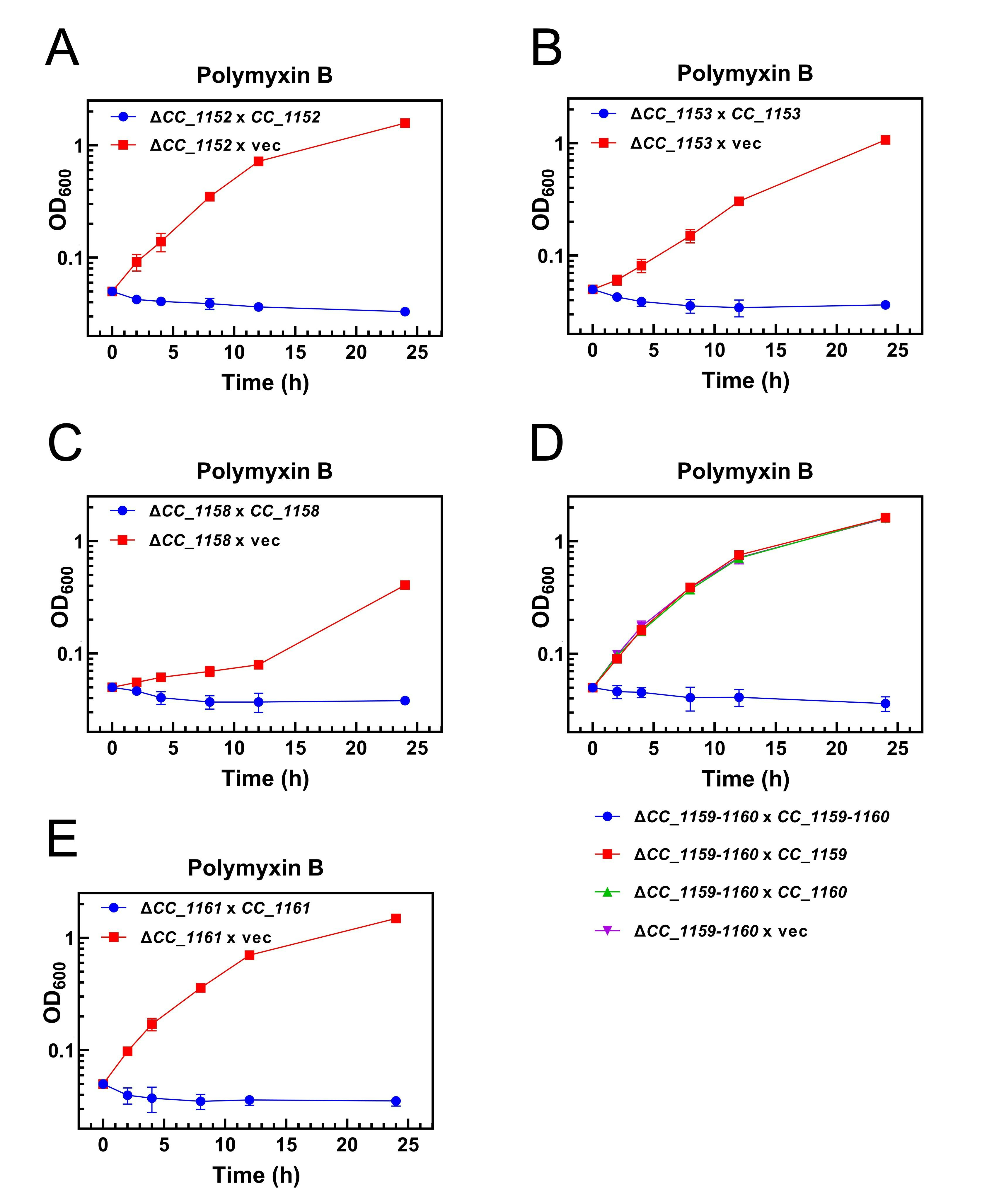

Supplement: S5 Fig — Growth (OD600) of C. crescentus mutants deficient in CC_1152 harboring the CC_1152-expressing plasmid (Δ1152 x 1152) or the empty vector pBXMCS-2 (Δ1152 x vec) (A), mutants deficient in CC_1153 harboring the CC_1153-expressing plasmid (Δ1153 x 1153) or the empty vector pBXMCS-2 (Δ1153 x vec) (B), mutants deficient in CC_1158 harboring the CC_1158-expressing plasmid (Δ1158 x 1158) or the empty vector pBXMCS-2 (Δ1158 x vec) (C), mutants deficient in CC_1159–1160 harboring the CC_1159-1160-expressing plasmid (Δ1159–1160 x 1159–1160), the CC_1159-expressing plasmid (Δ1159–1160 x 1159), the CC_1160-expressing plasmid (Δ1159–1160 x 1160), or the empty vector pBXMCS-2 (Δ1159–1160 x vec) (D), and mutants deficient in CC_1161 harboring the CC_1161-expressing plasmid (Δ1161 x 1161) or the empty vector pBXMCS-2 (Δ1161 x vec) (E). Data and bars represent the average and standard errors obtained from at least three independent experiments. Note that growth curves for strains Δ1159–1160 x 1159, Δ1159–1160 x 1160, and Δ1159–1160 x vec overlap in (D). (TIF) [file ppat.1012401.s011.tif]

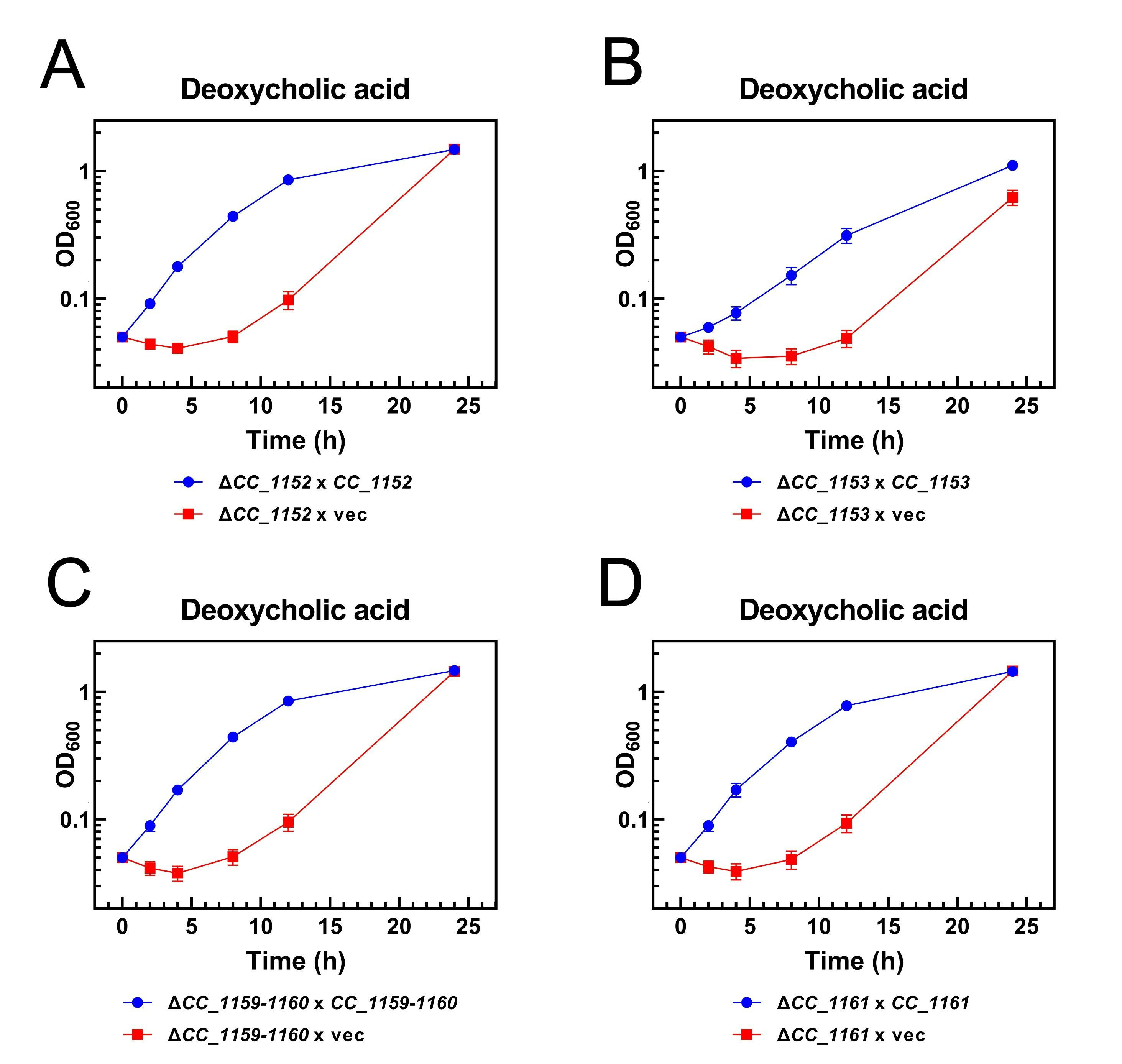

Supplement: S6 Fig — Growth (OD600) of different strains of C. crescentus [mutant SPG14 deficient in CC_1152 expressing intact CC_1152 in trans (Δ1152 x 1152) or mutant harboring the empty vector (Δ1152 x vec)(A), mutant SPG15 deficient in CC_1153 expressing intact CC_1153 in trans (Δ1153 x 1153) or mutant harboring the empty vector (Δ1153 x vec)(B), mutant SPG09 deficient in CC_1159/CC_1160 expressing intact CC_1159/CC_1160 in trans (Δ1159–1160 x 1159/1160) or mutant harboring the empty vector (Δ1159–1160 x vec)(C), and mutant SPG18 deficient in CC_1161 expressing intact CC_1161 in trans (Δ1161 x 1161) or mutant harboring the empty vector (Δ1161 x vec)(D)] was determined at 30°C on complex medium in the presence of deoxycholate (1 mg/ml). Data and bars represent the average and standard errors obtained from at least three independent experiments. (TIF) [file ppat.1012401.s012.tif]

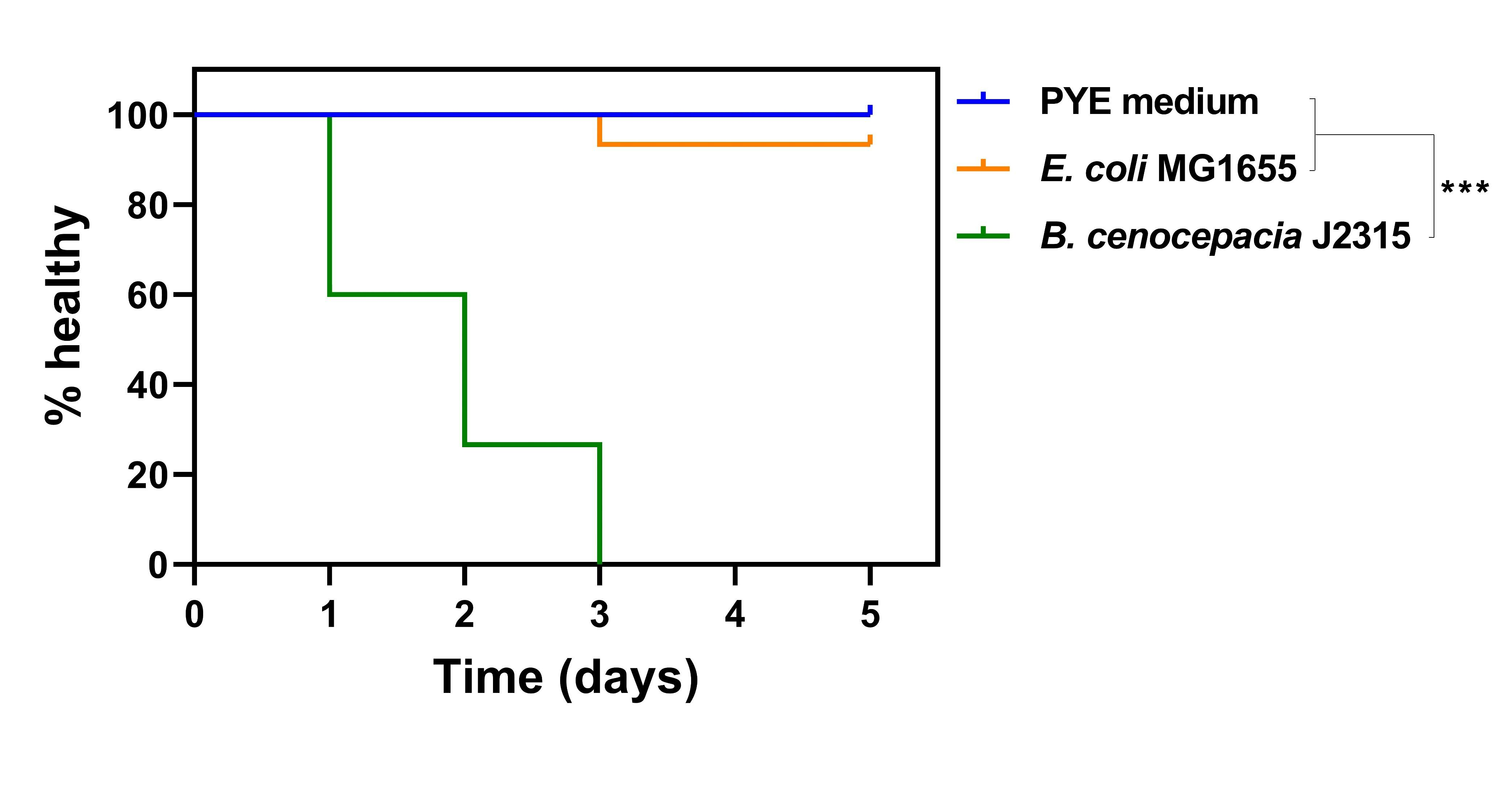

Supplement: S7 Fig — Survival curves are a representative cohort (n = 15) of at least three biological replicates (Mantel-Cox test for statistics, ***P<0.001). (TIF) [file ppat.1012401.s013.tif]

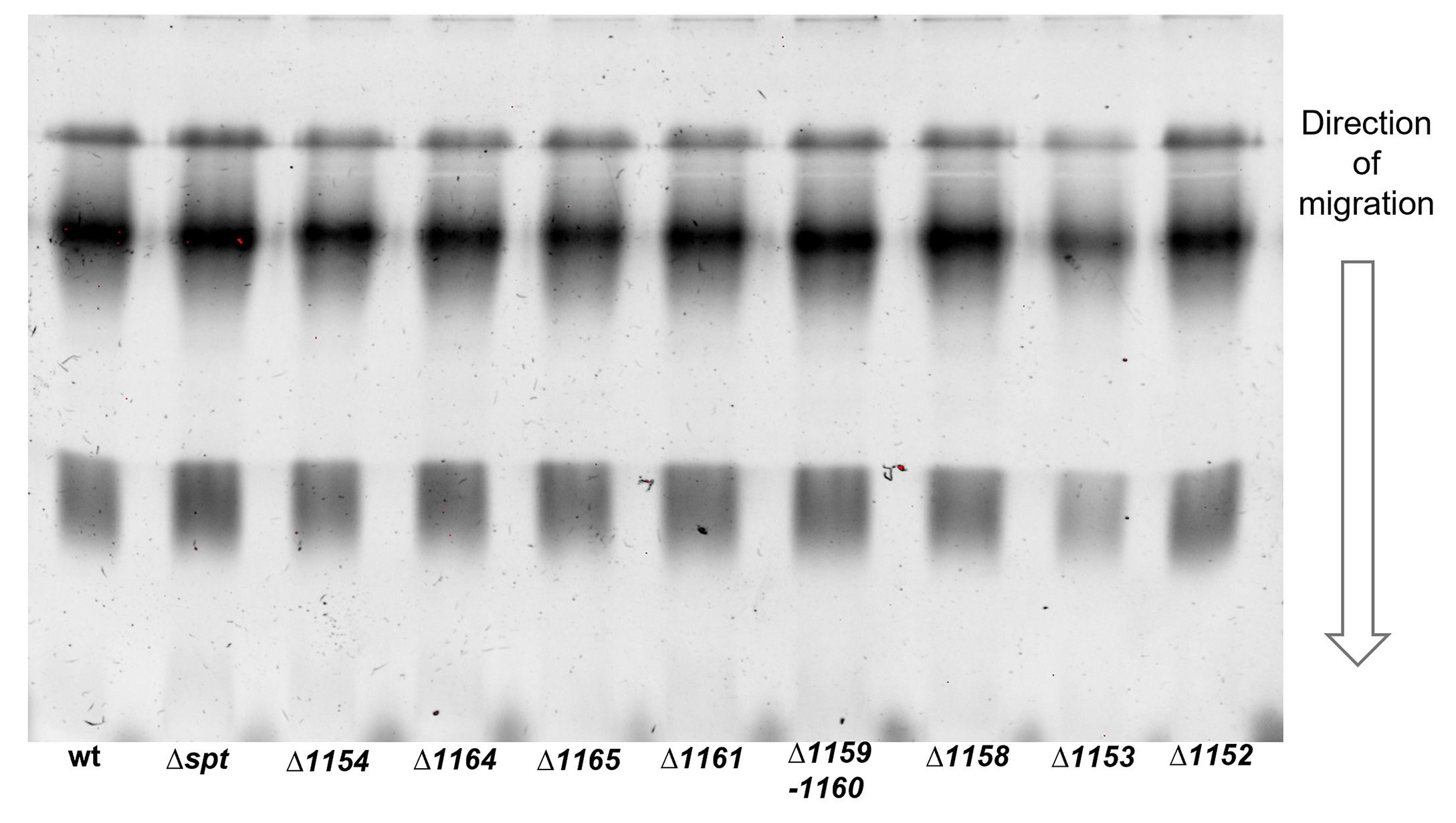

Supplement: S8 Fig — Hot aqueous-phenol LPS extractions were performed exactly as described [14] from 1 ml of exponentially growing cultures (OD600 = 0.8) of C. crescentus wild type (wt) and mutants affected in ceramide formation (Δspt, Δ1154, Δ1164, Δ1165) or in further conversion of ceramide to CPG2 (Δ1161, Δ1159/1160, Δ1158, Δ1153, Δ1152). Samples, corresponding to 1/40 of the original culture, were analyzed by gel electrophoresis (16.5%) in a Mighty Small II equipment (Hoefer). Carbohydrates were stained using Pro Q Emerald 300 Lipopolysaccharide Gel Stain Kit (Molecular Probes; P20495) per manufacturer´s instructions. (TIF) [file ppat.1012401.s014.tif]

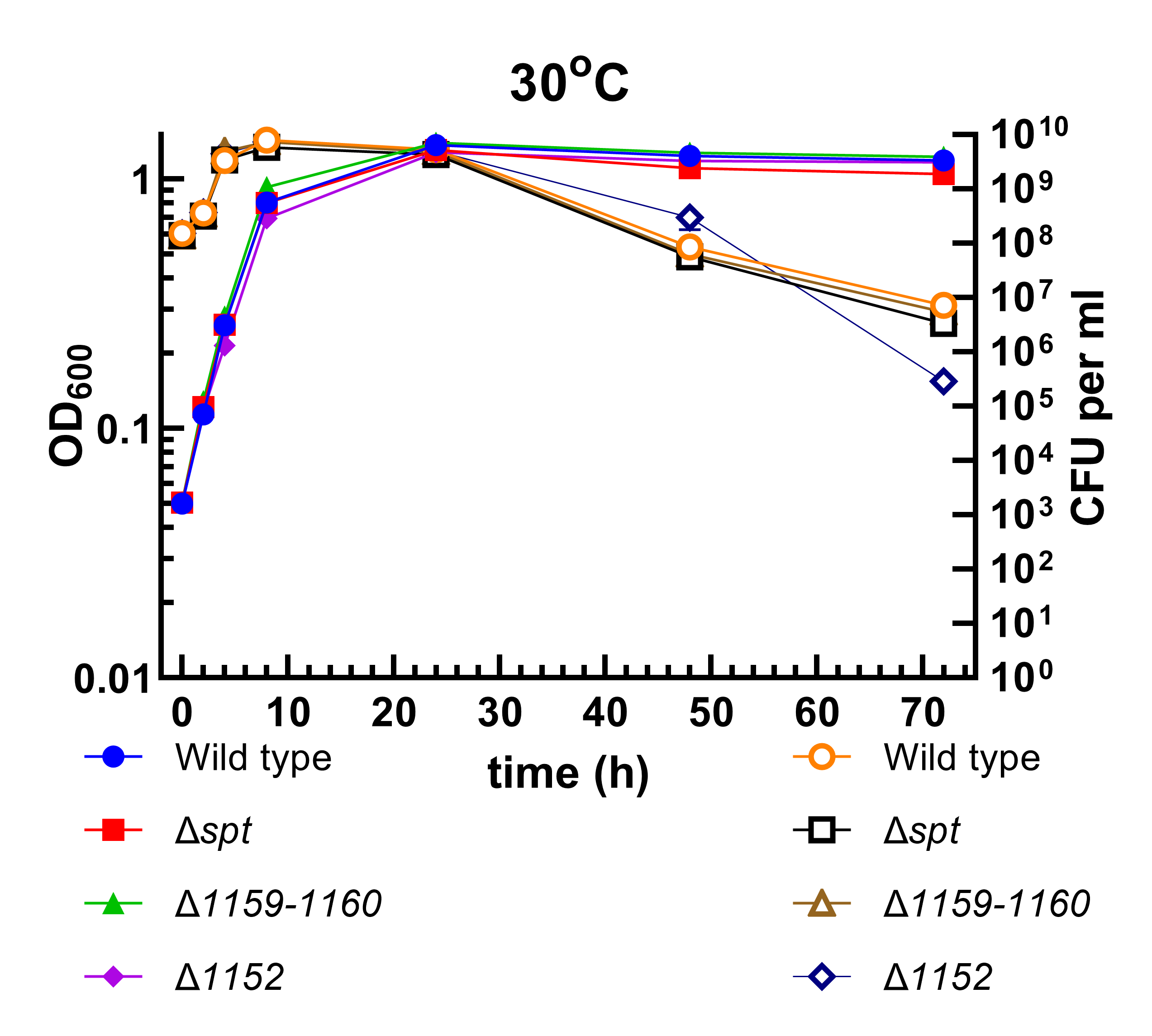

Supplement: S9 Fig — Growth and survival of wild type, spt-deficient mutant DAGS01 (Δspt), CC_1159/CC_1160-deficient mutant SPG9 (Δ1159/1160), or CC_1152-deficient mutant SPG14 (Δ1152) was determined at 30°C on complex medium. Growth of the C. crescentus strains was followed by measuring OD600 (filled symbols) whereas survival was quantified by determining CFU per ml (open symbols). Data and bars represent the average and standard errors obtained from at least three independent experiments. (TIF) [file ppat.1012401.s015.tif]
